# Alterations in the Ca^2+^ toolkit in oesophageal adenocarcinoma

**DOI:** 10.37349/etat.2021.00063

**Published:** 2021-12-31

**Authors:** Alana L. Cutliffe, Sharon L. McKenna, Darshan S. Chandrashekar, Alvin Ng, Ginny Devonshire, Rebecca C. Fitzgerald, Tracey R. O’Donovan, John J. Mackrill

**Affiliations:** 1 Department of Physiology, University College Cork, BioSciences Institute, T12 YT20 Cork, Ireland; 2 Cancer Research, UCC, Western Gateway Building, University College Cork, T12 XF62 Cork, Ireland; 3 Department of Pathology, Molecular & Cellular, University of Alabama at Birmingham, Birmingham, AL 35233, USA; 4 Cancer Research UK Cambridge Institute, University of Cambridge Li Ka Shing Centre, Robinson Way, CB2 0RE Cambridge, UK; The University of Texas at Arlington, USA

**Keywords:** Oesophageal adenocarcinoma, Ca^2+^ toolkit, acid-sensing, voltage-gated Ca^2+^ channel subunits, junctophilin 1, acid-sensing ion channel 4, transient receptor potential ion channel melastatin 5, secretory pathway Ca^2+^ ATPase 2

## Abstract

**Aim:**

To investigate alterations in transcription of genes, encoding Ca^2+^ toolkit proteins, in oesophageal adenocarcinoma (OAC) and to assess associations between gene expression, tumor grade, nodal-metastatic stage, and patient survival.

**Methods:**

The expression of 275 transcripts, encoding components of the Ca^2+^ toolkit, was analyzed in two OAC datasets: the Cancer Genome Atlas [via the University of Alabama Cancer (UALCAN) portal] and the oesophageal-cancer, clinical, and molecular stratification [Oesophageal Cancer Clinical and Molecular Stratification (OCCAMS)] dataset. Effects of differential expression of these genes on patient survival were determined using Kaplan-Meier log-rank tests. OAC grade- and metastatic-stage status was investigated for a subset of genes. Adjustment for the multiplicity of testing was made throughout.

**Results:**

Of the 275 Ca^2+^-toolkit genes analyzed, 75 displayed consistent changes in expression between OAC and normal tissue in both datasets. The channel-encoding genes, *N*-methyl-*D*-aspartate receptor 2D (*GRIN2D*), transient receptor potential (TRP) ion channel classical or canonical 4 (*TRPC4*), and TRP ion channel melastatin 2 (*TRPM2*) demonstrated the greatest increase in expression in OAC in both datasets. Nine genes were consistently upregulated in both datasets and were also associated with improved survival outcomes. The 6 top-ranking genes for the weighted significance of altered expression and survival outcomes were selected for further analysis: voltage-gated Ca^2+^ channel subunit α 1D (*CACNA1D*), voltage-gated Ca^2+^ channel auxiliary subunit α2 δ4 (*CACNA2D4*), junctophilin 1 (*JPH1*), acid-sensing ion channel 4 (*ACCN4*), *TRPM5*, and secretory pathway Ca^2+^ ATPase 2 (*ATP2C2*). *CACNA1D*, *JPH1*, and *ATP2C2* were also upregulated in advanced OAC tumor grades and nodal-metastatic stages in both datasets.

**Conclusions:**

This study has unveiled alterations of the Ca^2+^ toolkit in OAC, compared to normal tissue. Such Ca^2+^ signalling findings are consistent with those from studies on other cancers. Genes that were consistently upregulated in both datasets might represent useful markers for patient diagnosis. Genes that were consistently upregulated, and which were associated with improved survival, might be useful markers for patient outcome. These survival-associated genes may also represent targets for the development of novel chemotherapeutic agents.

## Introduction

Globally, oesophageal malignancies are the sixth-leading cause of cancer-related mortality [1]. The age-standardized 5-year net survival for oesophageal cancers (OCs, 2010–2014) was 21.9% for Ireland [2], 16.2% for the UK [2], and 20% for the United States [3]. Causes of poor prognosis include late diagnosis, incomplete resection of tumors, and resistance to chemotherapeutic and radio-therapeutic interventions [4]. Histologically, there are two major distinct forms of OC: oesophageal squamous cell carcinoma (OSCC), derived from epithelial cells, and oesophageal adenocarcinoma (OAC), arising from glandular cells [5]. OSCC is most prevalent in Southeast Africa and Asia [5]. By contrast, OAC is the most prevalent form in North America and Europe [5]. Risk factors for OAC include obesity, Barrett’s esophagus (BO, the replacement of normal, squamous epithelia with metaplastic columnar epithelia [6]), and gastroesophageal reflux disease (GORD) [1–7]. Two stomach-derived stimuli impacting oesophageal cells, because of GORD, are hydrochloric acid and bile acids (BAs) [8–9]. Little is known, however, about how oesophageal cells detect and respond to these stimuli [10].

**Figure 1.**
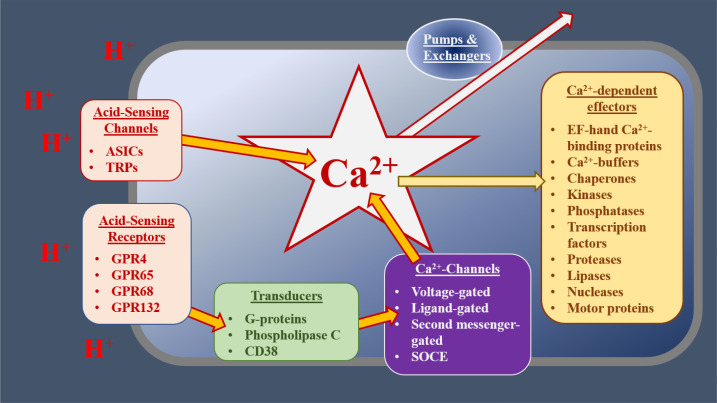
The potential role of the Ca^2+^ toolkit in OAC cells. At the cell surface, extracellular cues, such as decreases in extracellular pH (increased [H^+^]), are detected by receptors or cation channels, which gate to allow Ca^2+^ influx. GPRs activate transducers, such as G_αq/11_, which stimulate phospholipase C (PLC) and subsequently increase the concentration of IP_3_. This increased IP_3_ concentration, in turn, gates IP_3_Rs, releasing Ca^2+^ from the ER. Other Ca^2+^ channels and receptors respond to distinct stimuli or modify the signals generated by acid-sensing mechanisms. Elevated [Ca^2+^]_c_ alters the activities of various downstream effectors, resulting in the modification of cell physiology. Normal [Ca^2+^]_c_ is restored by buffers, pumps, and exchangers, operating in distinct subcellular compartments. Together, these receptors, Ca^2+^ channels, transducers, transporters, and effectors act as a Ca^2+^ toolkit, the components of which are distinctive for the type and condition of cells. ASICs: acid-sensing ion channels; GPR: G-protein coupled receptors; CD38: cluster of differentiation 38

Intracellular calcium (Ca^2+^) is a key second messenger in the cell [11–13]. In response to both extracellular and intracellular cues, cytoplasmic free Ca^2+^ [Ca^2+^]_c_ can be increased by up to two orders of magnitude [14, 15]. Such [Ca^2+^ ]_c_ transients regulate almost every aspect of cell biology including cell motility, gene expression, and cell death [11–15]. These increases in [Ca^2+^]_c_ can occur through several different mechanisms [15, 16]. [Ca^2+^]_c_ can be increased through the gating of ion-channel proteins, located in the plasma membrane (PM) or intracellular organelles, allowing the influx or release of Ca^2+^ into the cytoplasm [11]. These channels include those gated by changes in membrane potential [voltage-gated Ca^2+^ channels (VGCCs)], by ligands, by second messengers, by multiple stimuli [such as transient receptor potential (TRP) channels] or by the depletion of intracellular Ca^2+^ stores [store-operated Ca^2+^-entry (SOCE) channels, including Orai channels, which are gated by interactions with stromal interaction molecule (STIM) proteins] [11, 14, 15, 17]. Intracellular Ca^2+^ stores, such as the endoplasmic reticulum (ER) and sarcoplasmic reticulum (SR), act as reservoirs of Ca^2+^; this Ca^2+^ is released via channels such as the inositol 1,4,5-trisphophate (IP_3_) receptors (IP_3_Rs) and ryanodine receptors (RyRs) [11, 14, 15, 17]. Golgi, secretory pathway Ca^2+^ ATPase (SPCA) 1 and 2, and the SR/ER Ca^2+^-ATPases (SERCAs 1–3) are pumps that actively accumulate Ca^2+^ into intracellular stores, thereby decreasing [Ca^2+^]_c_ [11, 14, 15, 17]. [Ca^2+^]_c_ is also buffered by mitochondria, whose metabolic activities and effects on cell death are influenced by this second messenger [18]. As such, Ca^2+^ signalling can be influenced by mitochondrial proteins like those comprising the permeability transition pore, the mitochondrial Ca^2+^ uniporter (MCU), and mitochondrial Ca^2+^ exchangers [19, 20]. Changes in [Ca^2+^]_c_ are detected by effector proteins, including Ca^2+^-sensors, enzymes, transcription factors, and motor proteins [12, 13]. Together, the Ca^2+^-regulating and -sensing proteins within a cell can be considered a “toolkit”, which orchestrates stimulus-response coupling, Figure 1.

Altered Ca^2+^ signalling can result in pathological or pre-pathological states [12–17]. In cancer cells, the Ca^2+^ toolkit is remodeled to enhance the hallmarks of malignancy. This remodeling can impact proliferation, metastasis, or the evasion of apoptotic cell death [21]. Remodeled mitochondrial proteins, in particular, play a role in cancer cell proliferation and the evasion of cell death [22–27]. Ca^2+^ signalling has been shown to play critical roles in the biology of both normal and malignant oesophageal cells [21]. Extracellular Ca^2+^, for example, promotes the proliferation of human esophageal epithelial cell line (HET-1A) cells [28]. Alteration of the Ca^2+^ toolkit has been reported to contribute to the tumorigenic phenotype of other cancers, including OSCC [15, 17, 29–41]. Most studies to date have focused on the Ca^2+^ channel, TRP vanilloid 6 (TRPV6) [17, 34–40]. This channel was reported to be upregulated in breast, colon, ovary, prostate, and thyroid carcinomas [36]. In OSCC, however, TRPV6 was reported to be downregulated [35]. Increased *TRPV6*-expression has been associated with a better prognosis in cervical carcinoma [37]. The opposite effect (worsened survival with increased expression) has been reported in pancreatic cancer [38] and high-grade prostate cancer [34]. A sex-specific effect was noted in OSCC: male patients with *TRPV6*-downregulation had a poor 3-year disease-specific survival, whereas female counterparts showed enhanced survival [35]. Several TRPV6-related mechanisms have been proposed. *In vitro* analysis showed that *TRPV6*-silencing reduced breast cancer cell proliferation and promoted apoptosis [39]. In prostate-adenocarcinoma cells, TRPV6 supported high proliferation rates by providing constitutive Ca^2+^-influx for subsequent downstream activation of the nuclear factor of activated T cells (NFAT) [40]. *TRPV6*-expression is promoted by the activation of vitamin D_3_, estrogen and androgen receptors [36]. Such an interaction explains, at least in part, the effect of TRPV6 on the hormone-related cancers of the breast and prostate, as well as the sex-specific association of TRPV6 with OSCC survival [36]. Finally, the Orai3 protein works in conjunction with TRPV6 to promote proliferation in prostate and breast cancer [17]. Some study has been carried out on the TRPV2 channel, a channel that belongs to the same family as TRPV6. Patients with OSCC, gastric cancer, triple-negative, breast cancer, and bladder cancer, who had high *TRPV2*-expression, displayed poorer survival [29–31, 41]. Similar to TRPV6 and TRPV2 (poor prognosis with high expression in numerous cancers), high expression of the PM Ca^2+^ ATPase 2 (PMCA2) conferred resistance to apoptosis and was associated with a poor prognosis [32]. Other Ca^2+^-toolkit proteins implicated in various cancers include calcineurin, SERCA pumps, SPCAs, PMCAs, the IP_3_R, RyRs, STIM proteins, T-Type VGCCs, TRPC1, TRPC3, TRPC6, TRP ion channel melastatin 2 (TRPM2), TRPM7 and TRPM8 [15, 17, 34]. Some Ca^2+^-permeable channels have been implicated in the enhanced migration of various cancers: TRPC1, TRPM7, TRPM8, TRPV1, TRPV2, TRPV6, STIM1, Ca^2+^-release activated Ca^2+^ modulator 1 (Orai1) and some types of VGCCs [17, 33]. It is not known whether there are Ca^2+^-toolkit changes in OAC and if there is any association with tumor progression or patient survival.

Acidic conditions, occurring during GORD, are a key stimulus for the development of BO [6]; those with BO are 40–50 times more likely to develop OAC [42]. Decreased extracellular pH has been implicated in the proliferation of some cancer-derived cell lines (prostate, colon, lung, and breast cancers; pH ranged from 6.0–6.8) [43–48]. Extracellular acid (EA) has also been implicated in cancer metastasis in other cell lines (prostate, lung, and murine melanoma cancers; pH ranged from 5.9–6.8) [49–51]. Whether acidic extracellular environments result in oncogenesis in OAC, via the development of BO or by other mechanisms, remains unclear. Proposed oncogenic mechanisms include the production of reactive oxygen species (ROS), increased genomic instability, increased proliferation, dysregulation of apoptosis, and increased inflammation [43–56]. Exposure to EA has been linked to ROS production in BO [56]. Roesly et al. [52] showed that chronic exposure to BAs increased genomic instability and proliferation in BO and OAC cell lines. Indeed, the BA receptor farnesoid X receptor (FXR) is significantly overexpressed in BO (compared to normal mucosa, oesophagitis, and OAC) and may contribute to the regulation of apoptosis [53]. In nasopharyngeal carcinoma, BA-induced apoptosis (mediated by caspase-activated deoxyribonuclease) contributed to chromosomal rearrangements [54]. Inflammatory mediators [specifically: ROS, interleukin-1 (IL-1), IL-6, IL-8, and transforming growth factor-beta (TGF-β)], located in the oesophageal mucosa in GORD patients, have also been implicated in carcinogenesis [55]. Oxidative stress, caused by ROS, leads to DNA damage, RNA damage, activation of oncogenes, and inhibition of tumor-suppressor proteins [55]. IL-1, IL-6, and IL-8 enhance epithelial turnover (OAC is epithelia-derived cancer) [55]. TGF-β is generally anti-inflammatory [55]. TGF-β-responsiveness is reduced in OAC due to alterations in its signalling pathway [55]. Altered Ca^2+^ signalling could be contributing to all of these candidate mechanisms [57–69].

Multiple Ca^2+^-toolkit-related mechanisms link increased extracellular [H^+^] to increases in [Ca^2+^]_c_ and consequent changes in cell physiology [10, 12]. These include TRP channels [TRPA1, TRP ion channel classical or canonical 4 (TRPC4), TRPM2, TRPM5, TRPV1, TRPV4 and TRPV5]; GPRs, linked to PLC-activation and Ca^2+^-release via IP_3_Rs (GPR4, GPR65, GPR68 and GPR132); ASICs 1a, 1b, 2a and 3; vacuolar ATPases [ATPase H^+^ transporting V0 subunit A1 (ATP6V0A1), ATP6V0A2, ATP6V0A4, ATP6V0B and ATP6V0C]; proton exchangers [encoded by Solute Carrier 9A (SLC9A)1-9]; solute-carrier family 4 member A (SLC4A; SLC4A1-5 and SLC4A7-11); chloride-bicarbonate exchangers [solute-carrier family 26 member (SLC26; SLC26A and SLC26A1-10)]; carbonic anhydrases (CA; CA1-4 and CA6-14); and the hydrogen voltage-gated channel 1 (HVCN1) [10, 12, 70–78]. A broad overview of the role of some of these Ca^2+^-toolkit proteins in the cell has been detailed in Figure 1. Despite the potential impact of these acid-sensing proteins, their roles in the etiology of BO and OAC are currently unknown.

A limited number of studies have evaluated alterations of the Ca^2+^ toolkit, and their potential association with patient outcome, in OAC. Even less research has investigated how certain Ca^2+^-toolkit proteins may sense acidic environments and might, as a result, be remodeled to favor carcinogenesis. EA-exposure increased Ca^2+^-levels in an OAC cell line (pH 5) and in a murine, metastatic, melanoma cell line (pH 5.4-6.5) [60, 61]. Li and Cao [60] showed that the EA-stimulated increase in [Ca^2+^]_c_ in their OAC cell line activated the nicotinamide adenine dinucleotide phosphate (NADPH) oxidase 5-S enzyme, which subsequently elevated ROS and caused DNA damage.

In the current study, we examined the transcript levels of various components of the Ca^2+^ toolkit in OAC samples and in normal oesophageal samples, using data from both The Cancer Genome Atlas (TCGA) and the Oesophageal Cancer Clinical and Molecular Stratification (OCCAMS) consortium [79, 80]. We focused on a gene list of Ca^2+^-toolkit components, updated from an original review of these [13], with the addition of known components involved in acid-sensing [10] and those related to mitochondrial function [19, 20, 22–27].

Specifically, we aimed to:

i) Interrogate two distinct transcriptomic datasets to assess whether there is differential expression of any of 275 Ca^2+^-toolkit genes between OAC and normal tissue.
ii) Assess whether there is an association between the expression of Ca^2+^-toolkit genes and patient survival in OAC.
iii) Examine whether these survival-associated genes are associated with tumor grade or metastasis.

## Materials and methods

Two cancer transcriptome datasets were used in this study: TCGA (esophageal-carcinoma subset), accessed via the UALCAN portal [79, 81], and the OCCAMS dataset [80]. The TCGA dataset compared data from 89 OAC-tumor samples with 11 same-patient normal-adjacent tissue (NAT) samples. The OCCAMS dataset compared data from 213 OAC samples with data from 15 normal-tissue samples (from independent OAC cases).

The complete list of genes examined, and their associated proteins are detailed in Table S1. The expression levels of each of these 275 genes were assessed in OAC-tumor tissue and compared to expression levels in normal tissue. The mRNA expression levels of each gene were plotted as heat maps, using a log2 [transcripts per million (TPM) + 1] scaled look-up-table. To determine which genes were most consistently and significantly altered between normal and tumor tissue across both datasets, a dataset-specific weighted rank (the probability of altered expression of each gene, compared to normal tissue, relative to other differentially-expressed genes) was calculated for each gene and the average of both dataset-specific ranks was then taken. The association of each gene with patient survival was investigated using Kaplan-Meier survival analysis. This analysis was carried out initially on all genes in the OCCAMS dataset only; any genes with a statistically significant effect on survival were subsequently analyzed in UALCAN. A comparison was made between patients with high expression of the gene of interest (TPM reads above the upper quartile) and those with low expression (TPM below the lower quartile). To determine which survival-associated genes were selected for further analysis, the extent to which they were differentially expressed across both datasets was evaluated. Again, an average of the two dataset-specific weighted ranks was computed for each gene. Genes having an association with survival, which also had the smallest, average, weighted ranks (1 being the smallest and 75 being the largest) for differential expression, were selected for further analysis.

Selected genes were also examined for differential expression across OAC tumor grades and nodal metastatic stages. Tumour grade refers to changes in the morphology of cells, as assessed by microscopy [82]. Histological tumor grades in this analysis were stratified based on cellular differentiation: Grade 1 was well-differentiated; Grade 2 was moderately differentiated, and Grade 3 was poorly differentiated. The metastatic stage refers to tumor location and whether there was any metastasis in the lymph nodes or in distant sites [82]. For the metastatic-stage boxplots, the following categories were used: N0 corresponded to the tissue having no regional lymph-node metastasis; N1 corresponded to the tissue having metastasis in 1 to 3 axillary lymph nodes; N2 corresponded to the tissue having metastasis in 4 to 9 axillary lymph nodes, and N3 corresponded to the tissue having metastasis in 10 or more axillary lymph nodes.

### Statistical analysis

Gene-expression data for tumor *versus* normal tissue were compared using Welch’s *t*-tests. Associations of transcript-level (upper quartile *versus* lower quartile) with patient survival were presented in Kaplan-Meier plots and were statistically compared using log-rank tests. Associations between transcript levels and differentiation grade or lymph-node metastasis were presented as boxplots, compared by analysis of variance (ANOVA) with Tukey *post hoc* tests. For statistical comparisons, *P*-values of less than 0.05 were considered significant. An adjustment for multiplicity of testing (MOT) was made (for both UALCAN and OCCAMS data) using the false discovery rate (FDR) method [83, 84], for both the Welch’s *t*-tests and Kaplan-Meier, log-rank tests. Tukey, honest-significant-differences, *post hoc* tests served as an appropriate adjustment for MOT for UALCAN and OCCAMS ANOVA tests.

## Results

Transcript levels of 275 genes, encoding components of the Ca^2+^ toolkit, were investigated in two OAC datasets. A total of 392 statistical tests (Welch’s *t*-tests, Kaplan-Meier, log-rank tests and ANOVA with Tukey *post hoc* tests) were carried out in the UALCAN portal and 638 in the OCCAMS dataset [data was not available for four genes in OCCAMS: G protein subunit gamma 7 (*GNG7*), mucolipin 1 (MCOLN1), JPH3, and sorcin (SRI)]. Initial survival analysis of 271 genes was carried out in the OCCAMS dataset (the dataset with the most statistical power). The resulting survival-associated genes significantly associated with survival were then analyzed in UALCAN. One hundred and forty-eight gene variables [gene variable being defined as “a gene and an associated test”, such as “voltage-gated Ca^2+^ channel auxiliary subunit α2 δ4 (*CACNA2D4*)-heat map” and “acid-sensing ion channel 4 (*ACCN4*)-survival”] were considered significant in the UALCAN portal. After adjustment for MOT, this number was reduced to 130 significant, gene variables. In the OCCAMS dataset, 233 gene variables were considered significant following adjustment for MOT.

### Expression analysis

Of the 275 *t*-tests carried out on mRNA-expression levels in tumor *versus* normal tissue, 136 were statistically significant in UALCAN; after adjustment for MOT, this number was reduced to 118 (42.9% of the genes in the UALCAN dataset). Of the 271 heat-map-related *t*-tests carried out in OCCAMS, 182 (67.2%) were considered statistically significant following adjustment for MOT. Seventy-five of these genes (75/118 in UALCAN and the same 75/182 in OCCAMS) were differentially expressed across both datasets: 68 were upregulated compared to normal tissue; 4 [homer scaffolding protein 2 (*HOMER2*), *CACNA2D3*, voltage-gated Ca^2+^ channel, L-type, β 4 subunit (*CACNB4*), and *SLC9A4*] were downregulated; and 3 [*SLC26A9*, voltage-gated Ca^2+^ channel auxiliary subunit gamma 4 (*CACNG4*), and two-pore segment channel 2 (*TPCN2*)] were differentially expressed in opposing directions in the two datasets.

**Figure 2.**
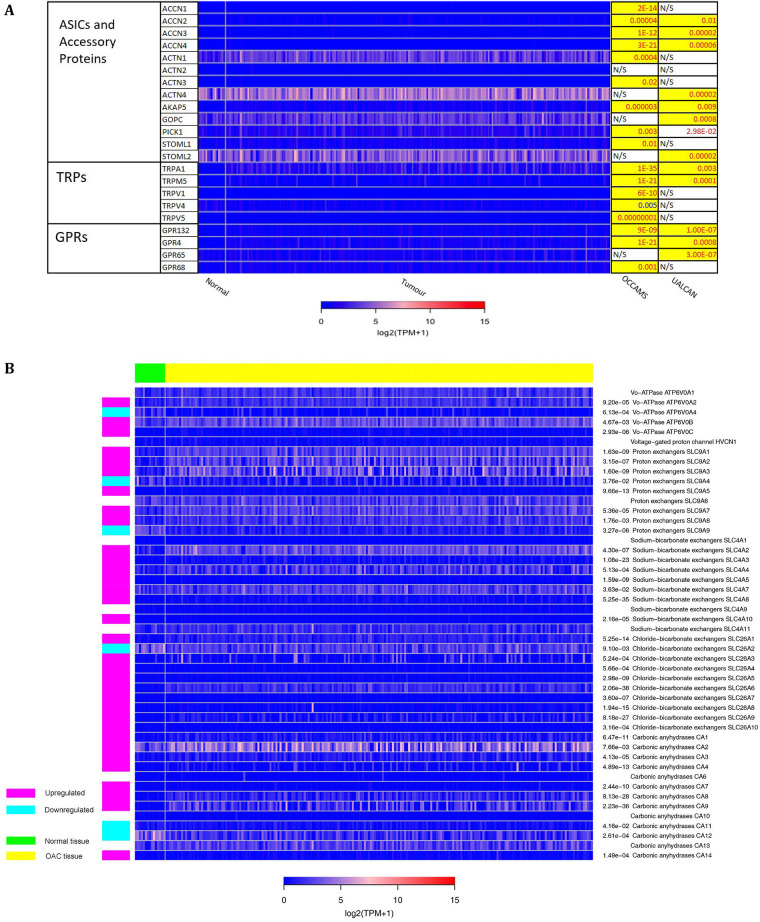
Expression in OAC of A) Acid-sensing channels, receptors and their accessory proteins; and B) proton-regulating proteins. Heat maps depicting the expression levels of genes encoding proteins involved in acid-sensing and proton homeostasis in OAC tumor *versus* normal tissue. The heat map shows expression data for patients from the OCCAMS dataset. The look-up table represents expression in TPM, with log2 (TPM + 1) scaling. The *P*-values for significant expression levels (significance was set at *P* < 0.05) from each dataset are listed on the right, with *P*-values in red font indicating upregulation, and *P*-values in blue font indicating downregulation, compared to normal tissue. The *P*-values highlighted in yellow were considered significant after adjustment for MOT. N/S indicates a non-significant change in expression levels in tumor *versus* normal tissue. GRPs: ground rubber particles; ACTN: actinin α; AKAP: A-kinase anchoring protein; GOPC: golgi-associated PDZ and coiled-coil motif-containing; PICK: protein interacting with C kinase; STOML: stomatin-like; NHE: Na-H exchange; NHX: Na^+^/H^+^ antiporte

**Figure 3.**
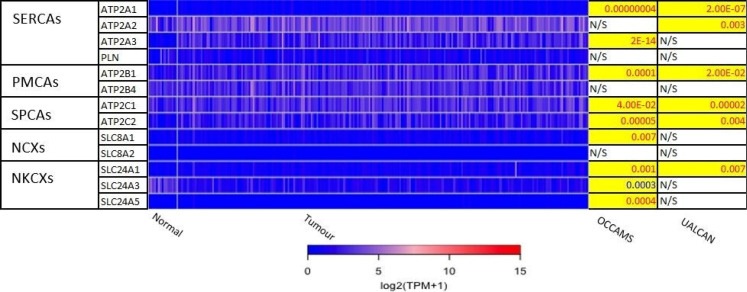
Expression of Ca^2+^ pumps and exchangers in OAC. Please see Figure 2 for details. NCXs: Na^+^/Ca^2+^ exchangers; NKCXs: sodium potassium calcium exchangers; ATP2A: sarco-endoplasmic-reticulum Ca^2+^ ATPase; PLN: phospholamban; ATP2B: PM Ca^2+^ ATPases; SLC8A: sodium-Ca^2+^ exchanger; SLC24A: sodium-potassium-Ca^2+^ exchanger

**Figure 4.**
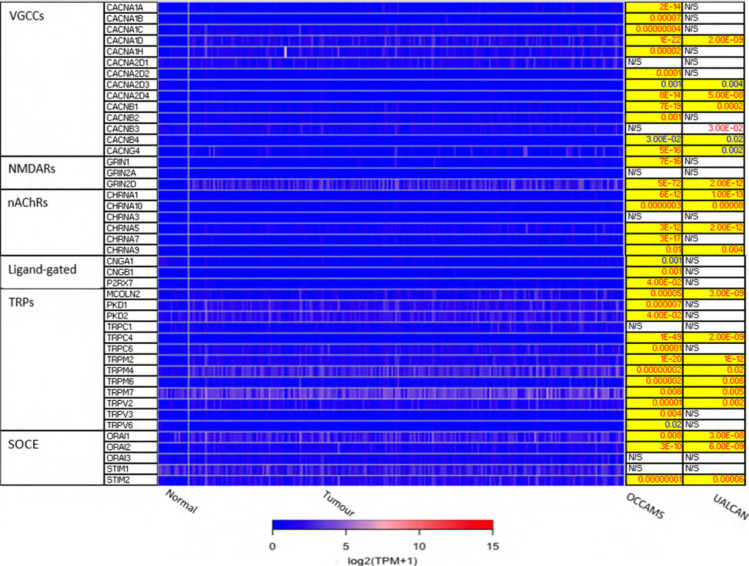
Expression of Ca^2+^ channels in OAC. *MCOLN1* was not available from the OCCAMS data. *MCOLN1* was upregulated, compared to normal tissue, in the UALCAN data (*P* = 9.95E-4). Please see Figure 2 for details. NMDARs: *N*-methyl-*D*-aspartate receptors; nAChRs: nicotinic acetylcholine receptors; CNGA: cyclic-nucleotide-gated channel subunit α; CNGB: cyclic-nucleotide-gated channel subunit β; P2RX7: purinergic receptor P2X, ligand-gated ion channel 7; PKD: polycystin; CHRNA: nAChR α

**Figure 5.**
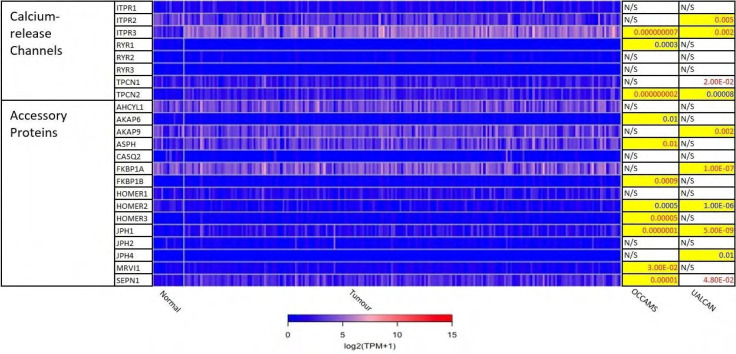
Expression of Ca^2+^-release channels and their accessory proteins in OAC. *JPH3* was not available from the OCCAMS data and was neither significantly upregulated nor downregulated in UALCAN. For details, please see Figure 2. ITPR: IP3 receptor gene; AHCYL: adenosylhomocysteinase-like; ASPH: aspartate β-hydroxylase; CASQ: calsequestrin; FKBP: FK506-binding protein; MRVI1: murine retrovirus-integration site 1 homolog; SEPN1: selenoprotein N1

The 271 genes presented in the heat-maps have been grouped into the following distinct functional categories: acid-sensing channels, receptors, and their accessory proteins; proton-regulating proteins (Figure 2A and B); Ca^2+^ pumps and exchangers (Figure 3); Ca^2+^ channels (Figure 4); Ca^2+^-release channels and their accessory proteins (Figure 5); transducers (Figure S1); mitochondrial-associated, Ca^2+^-toolkit genes (Figure S2); cytosolic Ca^2+^-sensors and -buffers (Figure S3); Ca^2+^-dependent chaperones (Figure S4); and Ca^2+^-dependent effectors (Figure S5).

Further analysis of the 75 genes (significantly differentially expressed across both datasets) revealed that expression of *N*-methyl-*D*-aspartate receptor 2D (*GRIN2D*), *TRPC4*, and *TRPM2* ranked the highest in terms of weighted significance. The individual *P*-values from the UALCAN data for these genes ranged from between to 2e-9 to 1e-12. The corresponding *P*-values from the OCCAMS dataset were: *GRIN2D*, 5e-72; *TRPC4*, 1e-49; and *TRPM2*, 1e-20.

### Survival analysis and grade and stage expression analysis

**Figure 6.**
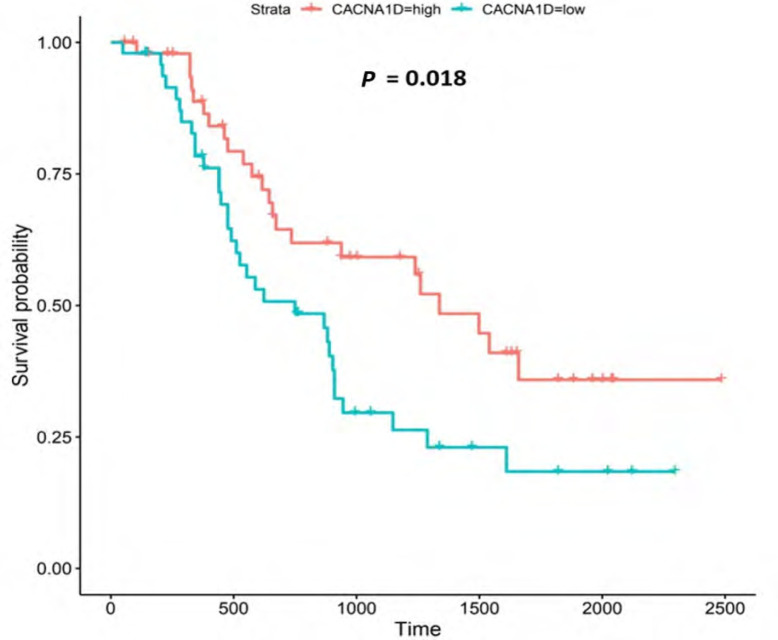
Kaplan-Meier survival plot for *CACNA1D*. Kaplan-Meier, survival plot (OCCAMS data) for *CACNA1D*. The comparison was made between patients with high *CACNA1D*-expression (TPM above the upper quartile) and those with low expression (TPM below the lower quartile). The plot shows survival probability with increasing time in days. A log-rank *P*-value of < 0.05 was considered statistically significant. An adjustment for MOT (FDR Method [83]) was made

**Figure 7.**
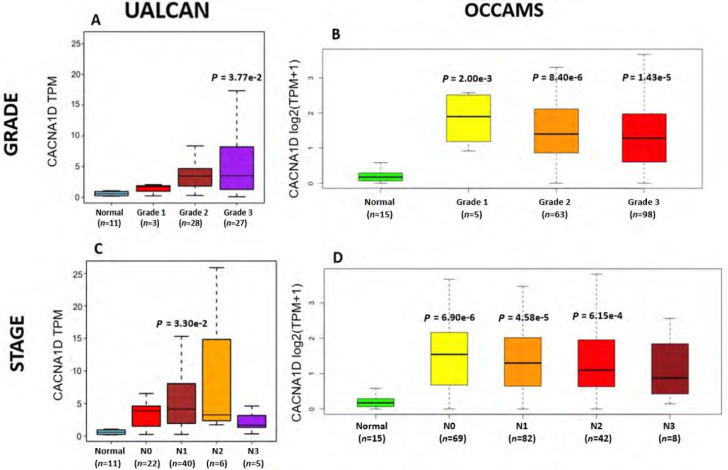
OAC tumor grade and nodal metastatic stage boxplots for *CACNA1D*. A). (UALCAN data) and B). (OCCAMS data) show *CACNA1D*-expression across various OAC-tumor grades, compared to normal tissue. C). (UALCAN data) and D). (OCCAMS data) show *CACNA1D*-expression across various nodal-metastatic stages, compared to normal tissue. A *P*-value of < 0.05 was considered statistically significant for the boxplots. Tukey HSD *post hoc* tests were adjusted for MOT. The number of tumor tissue, normal tissue (OCCAMS), or NAT (UALCAN) samples is represented by *n*

**Figure 8.**
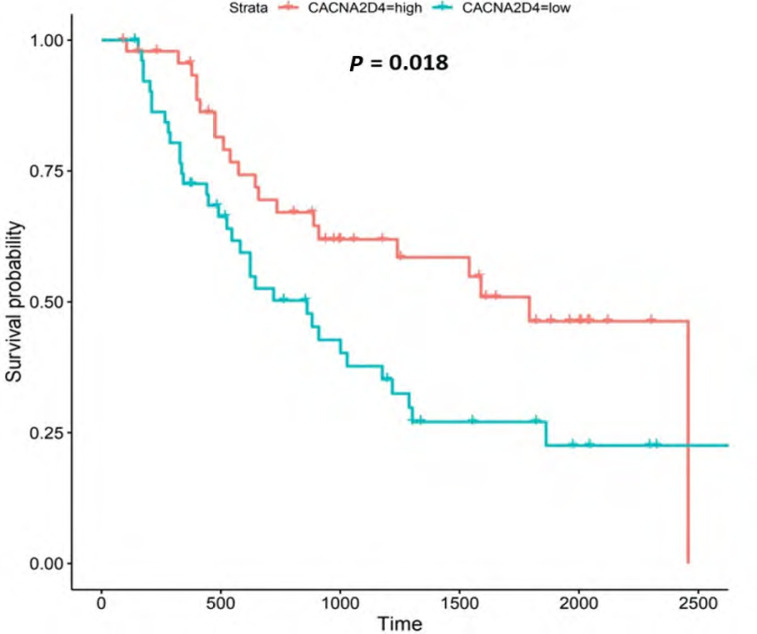
Kaplan-Meier survival plot for *CACNA2D4*. Details as described in Figure 6

**Figure 9.**
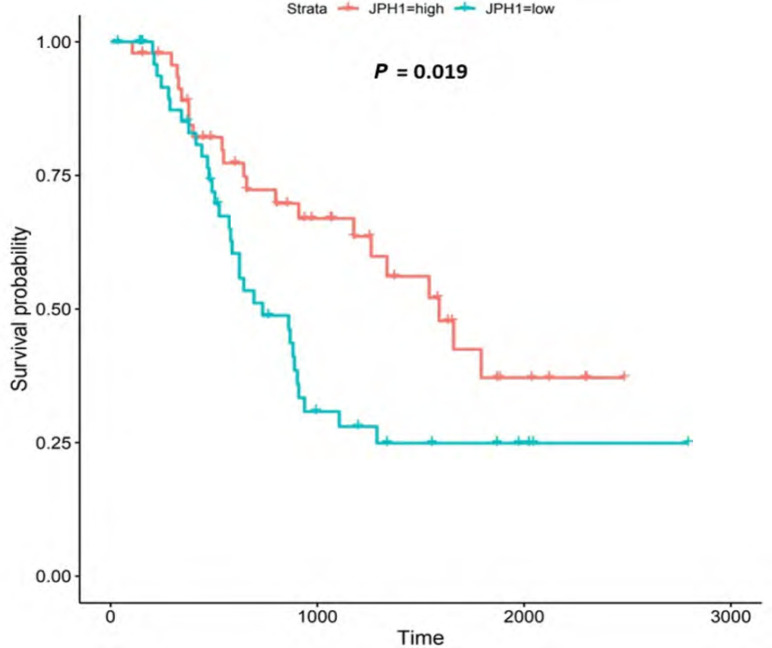
Kaplan-Meier survival plot for *JPH1*. Details as described in Figure 6

**Figure 10.**
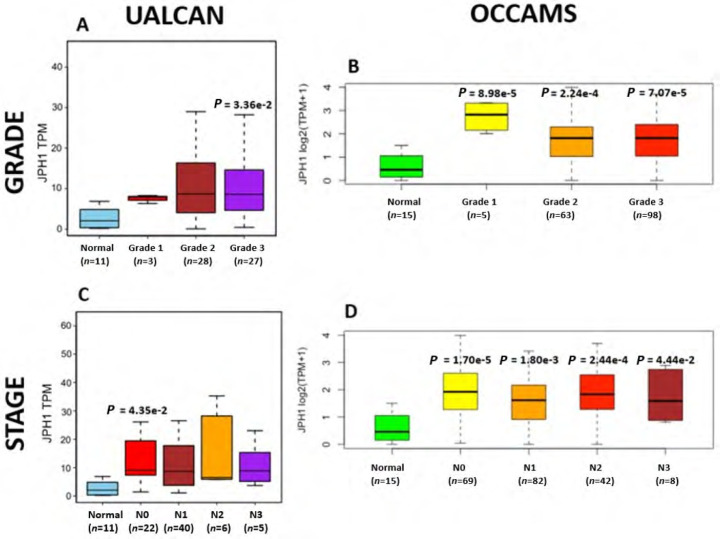
OAC tumor grade and nodal metastatic stage boxplots for *JPH1*. Details as described in Figure 7

**Figure 11.**
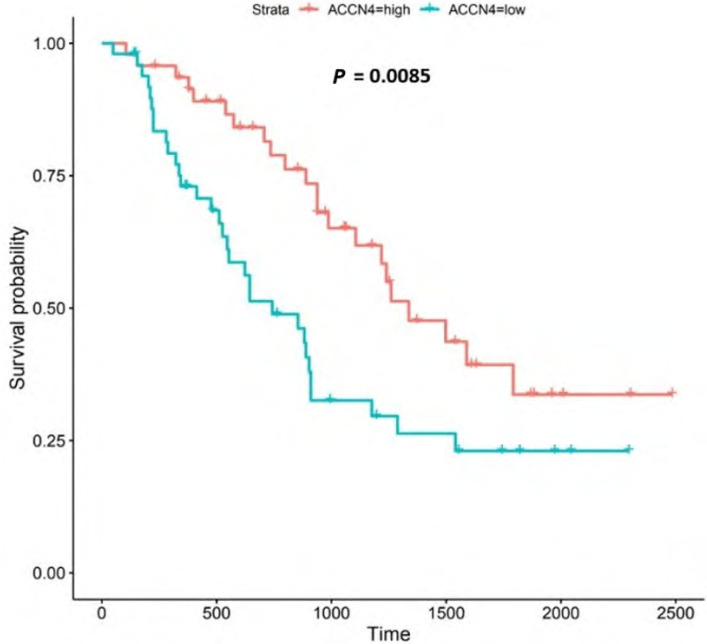
Kaplan-Meier survival plot for *ACCN4*. Details as described in Figure 6

**Figure 12.**
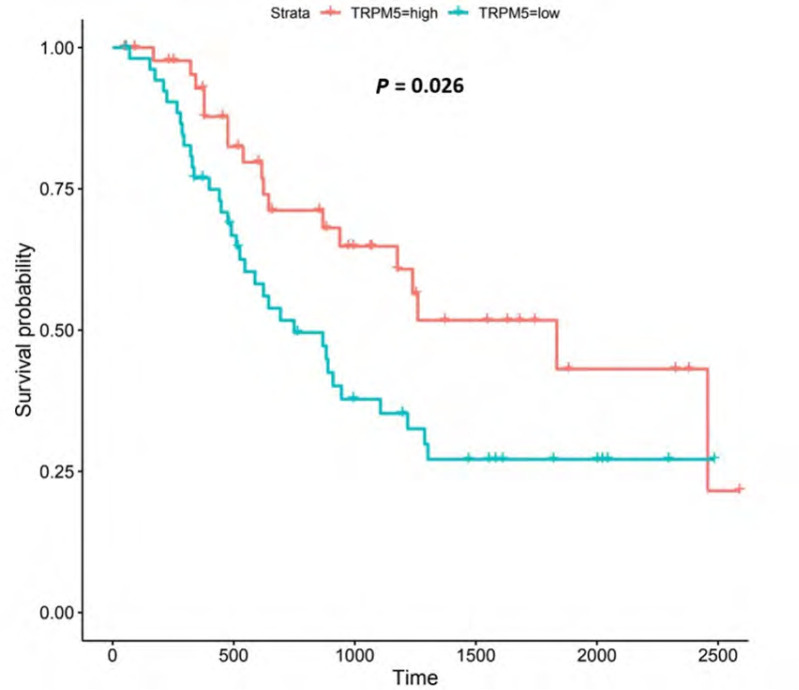
Kaplan-Meier survival plot for *TRPM5*. Details as described in Figure 6

**Figure 13.**
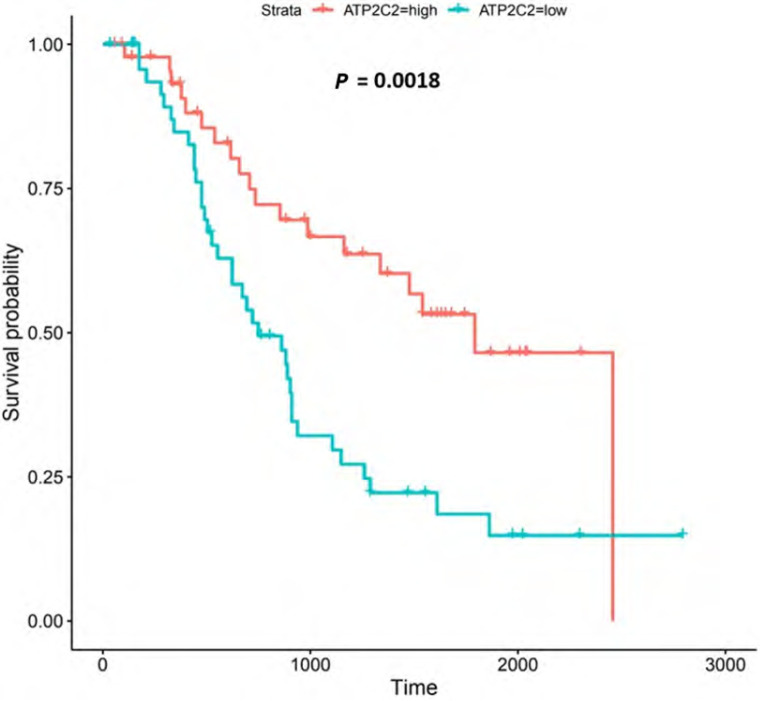
Kaplan-Meier survival plot for *ATP2C2*. Details as described in Figure 6

**Figure 14.**
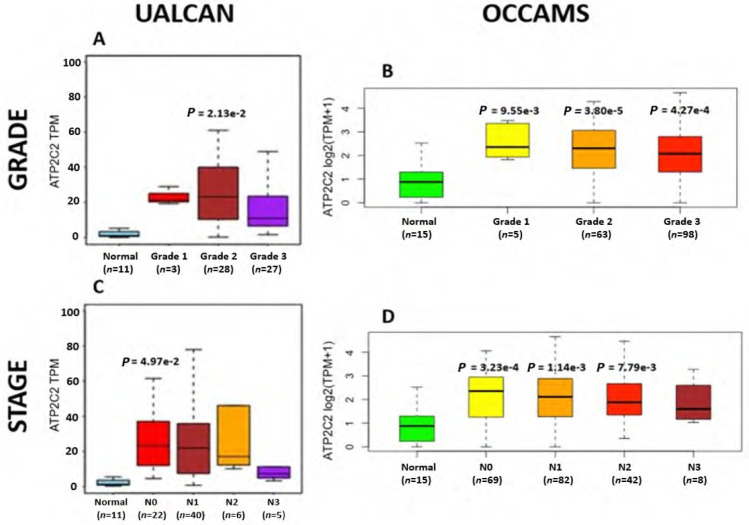
OAC tumor grade and nodal metastatic stage boxplots for *ATP2C2*. Details as described in Figure 7

Kaplan-Meier log-rank tests on the 271 genes from the OCCAMS dataset revealed that 21 had significant associations with survival (all were associated with improved survival with higher expression). These 21 genes were also analyzed using Kaplan-Meier log-rank tests in the UALCAN portal, but none proved significant. Of the 21 genes significantly associated with survival, 9 had significantly, altered, mRNA-expression levels (as assessed by weighted ranking) in both datasets. The top 6 of these 9 genes were shortlisted for further analysis. Listed in order of their statistical significance (weighted rank of mRNA-expression levels in both datasets), these survival-associated genes were: voltage-gated Ca^2+^ channel subunit α 1D (*CACNA1D*), *CACNA2D4*, junctophilin 1 (*JPH1*), *ACCN4*, *TRPM5*, and secretory pathway Ca^2+^ ATPase 2 (*ATP2C2*). Kaplan-Meier survival plots for these 6 genes are shown in Figures 6, 8, 9, 11-13, respectively. *CACNA1D*, *JPH1*, and *ATP2C2* were also consistently upregulated in various OAC grades and metastatic stages across both datasets. ANOVA boxplots for *CACNA1D*, *JPH1*, and *ATP2C2* are shown in Figures 7, 10, and 14, respectively. *CACNA2D4* was consistently upregulated in various metastatic stages across both datasets, but not in OAC grades (Figure S6). *ACCN4* was consistently upregulated in various OAC-tumor grades across both datasets, but not in OAC metastatic stages (Figure S7). *TRPM5* only showed significant upregulation in OAC-tumor grades and metastatic stages in the OCCAMS dataset (Figure S8). Despite the importance of the mitochondrion in Ca^2+^ signalling, we did not find any association of the 13 genes encoding mitochondrial proteins examined with survival outcomes.

#### CACNA1D

*CACNA1D* encodes the α_1D_ subunit of the L-type VGCC or Ca_v_1.3. The channel mediates the influx of Ca^2+^ into the cell, upon membrane depolarization [85]. It is found in smooth, muscle cells, skeletal muscle, ventricular myocytes, bone (osteoblasts), the brain, the kidneys, the pancreas, the ovaries, the retina, and the cochlea [85, 86]. Patients with higher *CACNA1D*-expression lived longer than those with lower expression levels (log-rank *P* = 0.018), Figure 6. *CACNA1D*-expression was upregulated in Grade 3 *versus* Normal (*P* = 3.77e-2) in the UALCAN portal and in Grades 1, 2, and 3 *versus* Normal in the OCCAMS dataset (*P* = 2.00e-3, *P* = 8.40e-6, and *P* = 1.43e-5, respectively), Figure 7. *CACNA1D*-expression was upregulated in the N1, nodal-metastatic stage *versus* Normal (*P* = 3.30e-2) in the UALCAN portal and in Stages N0, N1, and N2 in the OCCAMS dataset (*P* = 6.90e-6, *P* = 4.58e-5 and *P* = 6.15e-4, respectively), Figure 7.

#### CACNA2D4

*CACNA2D4* encodes the α2 and δ4 subunits of the L-type VGCC, Ca_v_1.4. Like Ca_v_1.3, the Ca_v_1.4 channel mediates the influx of Ca^2+^ into the cell upon membrane depolarization [87]. Patients with higher *CACNA2D4* expression lived longer than those with lower expression (log-rank *P* = 0.018), Figure 8.

#### JPH proteins

The JPH proteins contribute to [Ca^2+^]_c_ homeostasis by forming junctional membrane complexes [88]. Patients with higher *JPH1*-expression lived longer than those with lower expression (log-rank *P* = 0.019), Figure 9. *JPH1* was upregulated in various OAC-tumor grades in both datasets (Grade 3 *versus* Normal, *P* = 3.356e-2 for UALCAN and in Grades 1, 2 and 3 *versus* Normal, *P* = 8.98e-5, *P* = 2.24e-4 and *P* = 7.07e-5 for OCCAMS), Figure 10. JPH1 was upregulated in various OAC nodal-metastatic stages in both datasets (N0 *versus* Normal, *P* = 4.35e-2 for UALCAN and N0, N1, N2 and N3 *versus* Normal, *P* = 1.70e-5, *P* = 1.80e-3, *P* = 2.44e-4 and *P* = 4.44e-2 for OCCAMS), Figure 10.

#### ACCN4

*ACCN4* encodes the amiloride-sensitive, cation channel, ASIC4, which has been linked to synaptic transmission, pain perception, and mechano-perception [89, 90]. Patients with higher *ACCN4*-expression lived longer than those with lower expression (log-rank *P* = 0.0085), Figure 11.

#### TRPM5

TRPM5 is a member of the TRP superfamily of ion channels [91]. Patients with higher *TRPM5* expression lived longer than those with lower expression (log-rank *P* = 0.026), Figure 12.

#### ATP2C2

*ATP2C2* encodes an ATPase which transports Ca^2+^ and Mn^2+^ into the Golgi lumen to regulate protein sorting, processing, and glycosylation [92]. Patients with higher *ATP2C2*-expression lived longer than those with lower expression (log-rank *P* = 0.0018), Figure 13. *ATP2C2* was upregulated in various OAC-tumor grades in both datasets examined (Grade 2 *versus* Normal, *P* = 2e-2 for UALCAN and in Grades 1, 2 and 3 *versus* Normal, *P* = 9.6e-3, *P* = 3.8e-5 and *P* = 4.3e-4 for OCCAMS), Figure 14. *ATP2C2* was also upregulated in various nodal-metastatic stages in both datasets (N0 *versus* Normal, *P* = 5e-2 for UALCAN and in N0, N1 and N2 *versus* Normal, *P* = 3e-4, *P* = 1e-3 and *P* = 8e-3 for OCCAMS), Figure 14.

## Discussion

The goal of the present study was to examine whether there are alterations in the Ca^2+^ toolkit during the progression of OAC and if altered gene expression is associated with patient outcome. We particularly focused on Ca^2+^-toolkit proteins involved in acid-sensing and on Ca^2+^-toolkit proteins located in the mitochondrion. Ca^2+^ is a key second messenger which regulates most aspects of cell biology, including gene transcription, cellular secretion, cellular motility, and cell death [12]. In cancer cells, dysregulated Ca^2+^-signalling can lead to transformation, proliferation, and migration [21]. Increases in [Ca^2+^]_c_ can be elicited by decreases in extracellular pH [10]. During acid reflux, the pH of the distal esophagus can drop from about pH 6.5 to approximately pH 2 [93]. EA acts as a signal for the transformation of squamous to glandular cells in BO, a precursor to OAC [94]. The potential link between Ca^2+^ signalling and oncogenesis in OAC, and if such oncogenesis can be stimulated by EA, is largely unexplored and offers opportunities for the development of new chemotherapeutic approaches. Such strategies would target EA-related pathways in both pre-malignant disease and OAC.

The present transcriptomic analysis employed two different patient datasets. Our key findings are that *CACNA1D*, *CACNA2D4*, *JPH1*, *ACCN4*, *TRPM5*, and *ATP2C2* were significantly associated with improved survival and were also significantly upregulated compared to normal tissue in both datasets. Furthermore, there were significant differences in the transcription levels of *CACNA1D*, *JPH1*, and *ATP2C2* between various tumor grades and nodal-metastatic stages in both datasets. Finally, *GRIN2D*, *TRPC4*, and *TRPM2* were the most differentially expressed genes, based on weighted rank for significance-level, in both datasets.

*CACNA1D* encodes the α_1D_ subunit of the L-type VGCC, Ca_v_1.3. Currently, there is no evidence in the literaturelinking *CACNA1D* toacid-sensing. *CACNA1D*-expression was upregulated compared to normal tissue in both datasets investigated in the present study. Furthermore, high *CACNA1D*-expression was associated with improved survival outcomes. CACNA1D-expression was upregulated in Grade 3 *versus* normal tissue in the UALCAN portal and in Grades 1, 2, and 3 *versus* normal tissue in the OCCAMS dataset. *CACNA1D*-expression was upregulated in the second (N1) nodal-metastatic stage compared to normal tissue in the UALCAN portal and in the N0, N1, and N2 stages in the OCCAMS dataset. Other *CACNA1D*-expression-related studies have shown conflicting results on the effect of levels of this gene in cancers. Most studies have been carried out on prostate cancers, which contain the transmembrane protease, serine 2, gene-erythroblast transformation-specific-related gene (*TMPRSS2*-*ERG*) gene fusion. A bioinformatics meta-analysis of the Oncomine dataset (Wang et al. [95]) revealed that various subtypes of VGCCs (*CACNA1D* or Ca_v_1.3 included) were implicated in the development and progression of diverse types of cancer, including cancer of the prostate, breast, colorectum, bladder, stomach, lung, brain, uterus and esophagus [95]. The same study reported low *CACNA1D*-expression in sarcoma and renal tumors. Biasiotta et al. [96] noted that *CACNA1D* showed significantly increased expression in at least 13 of the 25 bladder-cancer datasets analyzed; these bladder-cancer results reinforce those from the meta-analysis by Wang et al. [95]. A Ca^2+^-toolkit-specific transcriptomic analysis (Pérez-Riesgo et al. [97]) revealed that *CACNA1D*-expression was increased 1.55-fold in human colorectal cancer cells, compared to normal colon cells. In endometrial carcinoma, *CACNA1D* was also upregulated [98]. In a study of radical prostatectomy patients, *CACNA1D*-expression was correlated with a higher Gleason score (a grading system for prostate cancer) and biochemical recurrence [99]. An analysis of the Oncomine dataset revealed that *CACNA1D*-expression was significantly higher in prostate cancers with the *ERG*-gene fusion, compared with the cases without this gene fusion [100]. Jhavar et al. [101] observed that *CACNA1D* was among the top ten differentially-expressed genes in the ERG-subtype of prostate cancer, compared to samples lacking ERG-expression. Setlur et al. [102] identified *CACNA1D* as part of an 87 gene signature for ERG-fusion-bearing prostate cancer. An epigenomic-profiling study of prostate cancer tumors noted that *CACNA1D* was among the top-ten-ranked differentially-methylated (hypomethylated) genes in tissues with ERG fusion, compared to those without [103]. *CACNA1D*, mRNA-expression was also inversely correlated with methylation of the gene [103]. Phan et al. [104] used Oncomine to calculate the changes in mRNA-expression of VGCCs in 20 types of cancer: in contrast to our findings, *CACNA1D* exhibited low expression in the brain, kidney, and lung tumors [104]. Analyses relating to the Ca_v_1.3 protein are consistent with our gene-expression findings. Fourbon et al. [105] demonstrated that the Ca_v_1.3 protein was more abundant in colorectal-cancer biopsies, compared to normal tissue. Chen et al. [100] noted that the Ca_v_1.3 protein was more abundant in prostate cancer and modulated androgen receptor transactivation. Furthermore, the use of Ca^2+^-channel blockers (dihydropyridines, phenylalkylamines, and benzothiazepines) was associated with a reduced risk for a higher Gleason score and ERG-positive prostate cancer [106]. Two studies focused on the association of *CACNA1D* with survival outcomes [107, 108]. Wang et al. [107] identified *CACNA1D* as part of an 18-ion-channel, prognostic-gene signature in glioma (the direction of the association with survival outcomes was not reported); this finding was observed even though *CACNA1D*-expression was downregulated in these cells. Xing et al. [108] noted that *CACNA1D* was one of 8 out of 3,747 differentially-expressed genes associated with survival outcomes in colon adenocarcinoma: consistent with our findings, those with higher *CACNA1D*-expression lived longer than those with lower expression [108]. Similar to our metastasis-related findings, *CACNA1D* was among the 6 genes associated with tumor node metastasis staging in colon adenocarcinoma [108]. Additionally, Fourbon et al. [105] showed that colon cancer cell migration was affected by *CACNA1D*-expression: the migration was decreased when *CACNA1D* was silenced.

The observation that *CACNA1D* was upregulated in OAC, but was also associated with improved patient survival, is of interest. Such associations with improved survival may be due to the potential effect of *CACNA1D* on Ca^2+^-dependent cancer-cell death. VGCC-associated (particular Ca_v_1-associated), Ca^2+^-dependent cell death has been well described in pancreatic β cells [109], but to date, not in OAC. Several cancer-predisposing mechanisms have been linked to VGCC function, including the direct influx of Ca^2+^ into the cell, the involvement of VGCC subunits, and the involvement of the steroid 17β-estradiol [98, 105, 110]. Ca^2+^ can enter the cytoplasm from the ER through the interaction between VGCCs and the RyR1, a process which facilitated by JPHs [111, 112]. The role of VGCC accessory subunits has been described in the mechanistic paragraph for *CACNA2D4* below. The potential role of 17β-estradiol in VGCC-associated carcinogenesis has been studied in endometrial carcinoma. Specifically, upregulation of *CACNA1D* was associated with increased proliferation and migration in endometrial carcinoma tissue. These effects were enhanced by 17β-estradiol, via the G protein-coupled estrogen receptor [98]. A previous study showed that these estrogen-stimulated effects were decreased by Ca^2+^-channel blockers (nifedipine and mibefradil) [110]. It is possible that any of the cancer-predisposing mechanisms of VGCCs mentioned could be inactivated in OAC, particularly if such inactivation was acid-dependent. Such inactivation would be consistent with the improved patient survival observed in the present study.

The *CACNA2D4* gene encodes the α2 and δ4 subunits of VGCC complexes and has been reported to be an oncogene [113]. *CACNA2D4*-expression was upregulated compared to normal tissue in both OAC datasets. High *CACNA2D4* expression was also associated with improved survival outcomes. *CACNA2D4* was upregulated compared to normal tissue in the fourth (N3), nodal-metastatic stage of OAC in the UALCAN portal, and in the first (N0) and third (N2) nodal-metastatic stages in the OCCAMS dataset. There is a paucity of literature investigating the role of *CACNA2D4* in OAC and in acid-sensing. A DNA-methylation study showed that *CACNA2D4* mRNA expression was upregulated in cultured gastric cancer cell lines, compared to normal stomach cells [87]. The role of *CACNA2D4* in pancreatic adenocarcinoma was examined by Xu et al. [114], using data from TCGA: in contrast to our findings, the authors noted a poor prognosis in a subset of patients with high *CACNA2D4*-expression. An analysis of 98 Ca^2+^-regulating genes from two gene-expression-profiling datasets on gastric cancer, highlighted that *CACNA2D4* was associated with either a 40% decrease (Dataset One) or a 2.9-fold increase (Dataset Two) in overall survival [41]. Similar to our findings, *CACNA2D4* was one of the few genes associated with the metastasis of uveal melanoma [115].

Interestingly, the related *CACNA2D3* gene (encoding α2 and δ3 subunits) was downregulated in OAC tissue compared to normal tissue, in both the UALCAN and OCCAMS datasets; we did not, however, identify an association between *CACNA2D3*-expression and survival. Like *CACNA2D4*, research into the role of *CACNA2D3* in OAC and in acid-sensing is lacking. However, there have been a number of studies on OSCC: Li et al. [116] reported tumor-suppressor activity of *CACNA2D3* in OSCC cell lines and demonstrated that decreased expression in OSCC patients was associated with poor survival and enhanced metastasis. Increased *CACNA2D3*-expression has also been linked with enhanced chemosensitivity of OSCC to cisplatin [117]. An association between *CACNA2D3* and both patient survival and tumor metastasis has been demonstrated in other cancers [87, 118]. High *CACNA2D3*-expression was associated with improved survival outcomes in advanced gastric cancer: patients with detectable *CACNA2D3* gene methylation had a significantly shorter survival time than patients without this methylation [87]. Methylation-dependent transcriptional silencing of *CACNA2D3* was shown to contribute to the metastatic phenotype of estrogen-receptor-positive primary breast cancer, again illustrating a protective nature of the gene [118]. We did not perform a metastasis-related analysis for *CACNA2D3* in our study because it did not show any significant association with survival. However, it could be useful to investigate whether *CACNA2D3* is a tumor suppressor in OAC. It would also be valuable to know whether there are any synergistic effects between *CACNA2D4* and *CACNA2D3*; the literature points to each gene having opposing roles in the cancers.

The observation that *CACNA2D4* upregulation was associated with improved patient survival warrants further study. The increased expression of VGCC accessory subunits, α_2_δ and β, has been related to different cancer hallmarks in liver, ovarian, prostate, pancreatic, lung, and colon tumors [119]. Supporting our observations, Wang et al. [113] showed that *CACNA2D4* played a role in mitigating the adverse effects of first-line chemotherapy (adriamycin or cisplatin) in the treatment of gastric cancers overexpressing bromodomain-containing protein 9 (BRD9). A mechanistic study has been carried out on *CACNA2D3*. Li et al. [116] demonstrated that *CACNA2D3* inhibited tumorigenicity by arresting the cell cycle at the G1/S checkpoint, through increased p21 and p53 expression. Li et al. [116] also demonstrated that *CACNA2D3* inhibited cell motility and induced Ca^2+^-dependent apoptosis. Similar to the *CACNA2D4* observations of Wang et al. [113], Nie et al. [117] noted that increased expression of *CACNA2D3* enhanced the chemosensitivity of OSCC to cisplatin via Ca^2+^-mediated apoptosis and the suppression of the phosphoinositide 3-kinase/protein kinase B (PI3K/Akt) pathway. The adverse effect of *CACNA2D3*-methylation (*CACNA2D3*-downregulation) has been described [87, 118]. Elucidating the effects of *CACNA2D4* on tumorigenicity, cell motility, apoptosis and chemosensitivity would be an invaluable addition to Ca^2+^ and OAC literature and would consolidate *CACNA2D3*-related literature.

*JPH1* encodes the JPH1 protein [4]. The JPH proteins contribute to [Ca^2+^]_c_ homeostasis by forming junctional membrane complexes; this is achieved by anchoring the Sarco-/Endoplasmic reticulum to the PM [4]. *JPH1* and *JPH2* are abundant in skeletal muscle and their suppression leads to the disruption of the activity of SOCE [88]. In our study, high *JPH1*-expression was associated with improved patient survival. Additionally, *JPH1*-expression was upregulated compared to normal tissue across both the UALCAN and OCCAMS datasets. Further analysis revealed that *JPH1* was upregulated across advanced OAC-tumor grades and OAC nodal-metastatic stages. There is no literature linking a role for *JPH1* in OAC or acid-sensing. *JPH1* was among the upregulated genes in an analysis of lung cancer [120]. Zou et al. [121] noted that the long non-coding form of *JPH1* RNA, Lnc-JPH1-7, was upregulated 35-fold in TCGA samples of head-and-neck, squamous-cell carcinoma. Low expression of this long non-coding RNA promoted survival, consistent with it suppressing the expression of the coding form of *JPH1* [121]. A study by Tsantoulis et al. [122] on uveal melanoma demonstrated that *JPH1*-expression was associated with relapse. By contrast, in uveal-melanoma tissue, JPH1 expression was downregulated compared to normal tissue [123]. An analysis by Que et al. [124] revealed that *JPH1* was one of 14 mRNA transcripts involved in regulating a microRNA-circRNA network of genes likely involved in the development or prevention of colorectal cancer [124]; whether *JPH1*-expression was upregulated or downregulated was not reported in this analysis. A mutation in the *JPH1* gene was noted in a patient with human T-lymphotropic virus type-1 (HTLV-1)-associated myelopathy/tropical spastic paraparesis who subsequently developed adult T-cell leukemia [125]. *JPH1* was among the top 20 genes associated with survival in endometrial carcinoma [126]; whether *JPH1* was associated positively or negatively with survival was not reported. Consistent with our findings, *JPH1* was one of 14 of 7,222 genes identified as being strongly associated with a better prognosis in squamous-cell lung carcinoma [127]. Again, Zou et al. [121] noted associations between elevated Lnc-JPH1-7-levels and head-and-neck squamous-cell carcinoma: this time a link with poor prognosis was observed. The authors also highlighted a significant correlation between Lnc-JPH1-7 and the advanced, tumor stage [121]. Metastasis-related literature also aligns with our findings. Tsantoulis et al. [122] demonstrated that the expression of a combination of *JPH1* and the protein-tyrosine phosphatase 4A3 (*PTP4A3*) gene correlated with an increased risk of developing liver metastasis in colorectal and breast cancer (hormone-positive tumors only) [122]. Zou et al. [121] illustrated how short hairpin RNA-mediated knockdown of Lnc-JPH1-7 reduced the expression of epithelial-mesenchymal-transition-promoting genes in head-and-neck squamous-cell carcinoma cell lines.

Similar to *CACNA1D* and *CACNA2D4*, high *JPH1*-expression was associated with improved patient survival in our OAC study. A specific microRNA, miR-145, is the most compelling proposed underlying mechanism for this association with survival. miR-145 has been shown toregulate tumorigenesis, proliferation, differentiation, apoptosis, metastasis, angiogenesis, and therapeutic resistance in certain cancers [128, 129]. It has also been downregulated compared to normal tissue in several cancers, including OSCC. If upregulated, as in the case of OAC, miR-145 is typically accepted as a tumor-suppressor and a suppressor of therapeutic resistance [129]. Xu et al. [128] noted that *JPH1* was one of 78, potential targets of miR-145. If in the present study, *JPH1* is upregulated in OAC and associated with improved survival, it would suggest that miR-145 is interacting with *JPH1* post-transcriptionally to favor tumor suppression. Although calmodulin-dependent protein kinase 1D (*CAMK1D*) and calmodulin-dependent protein kinase 2D (*CAMK2D*) were also targeted by miR-145 in the study by Xu et al. [128], these proteins exhibited little significance in the present study. Other studies have focused on *JPH2* and *JPH3*. *JPH2* was among 10 individual genes of a DNA-methylation signature associated with overall survival of gastric cancer patients [130]: in contrast to the JPH1 observations of the present study, higher *JPH2*-methylation (gene-downregulation) was associated with longer survival [130]. In lung adenocarcinoma, *JPH3* was downregulated 0.2-fold by *S100A2* (associated with favorable prognosis in p53-negative tumors) and 0.43-fold by *S100A4* (associated with poor prognosis in p53-positive tumors) [131]. The potential role of the upregulation of *S100A2* and *S100A4* in the downregulation of *JPH1* was not consistently observed in the present study. Again, laboratory experimentation is required to verify the roles of *JPH1* in OAC.

*ACCN4* encodes ASIC4 [6]. ASIC4 is thought to regulate other members of the ASIC family, particularly in the generation of pain-related currents [89]. ASICs 1a, 1b, 2a and 3 all sense transient and sustained acidification [90, 132]. In our study, we noted that *ACCN4* was upregulated in both the UALCAN and the OCCAMS datasets. High *ACCN4*-expression was associated with improved survival outcomes. Further analysis revealed that ACCN4 was upregulated compared to normal tissue in advanced OAC-tumor grades (both datasets), and in the first (N0) nodal-metastatic stage in the OCCAMS dataset. There is no literature linking *ACCN4*-expression with OAC. Other cancer-related literature focuses on *ACCN4*-expression and metastasis, the results of which are conflicting. A study by Marques et al. [133] found that *ACCN4* was upregulated by r1881 (a synthetic androgen) in hormone-therapy-resistant prostate cancer cell lines. By contrast, *ACCN4* was overexpressed in cisplatin-sensitive ovarian cancer cells [134], suggesting a protective role of the gene. *ACCN4*-downregulation in head-and-neck squamous-cell carcinoma was noted by Braakhuis et al. [135]. A polysaccharide from the marine algae, *Gracilariopsis lemaneiformis*, (known for its anticancer activity) significantly decreased *ACCN4* transcription in a lung-cancer cell line [136]. In an analysis of the Oncomine dataset, focusing on 5 histologically distinct solid tumors (bladder cancer, glioblastoma, melanoma, breast, invasive-ductal cancer, and lung carcinoma), *ACCN4*-expression was neither upregulated nor downregulated, compared to normal tissue [96]. There is a paucity of literature associating *ACCN4*-expression with survival outcomes. Two studies highlighted a role for *ACCN4* in metastatic tissue (one indicating a positive association and one indicating a negative association) [135, 137]. Di Pompo et al. [137] investigated whether breast cancer metastasis-induced, bone pain was associated with the effect of EA acting on the mesenchymal, tumor-associated stroma. The authors used human osteoblast primary cultures from healthy donors and cancer-associated fibroblasts from the tumor biopsies of patients with metastasis [137]. After exposure of both types of cells to a medium at pH 6.8 for 6 h, they noted increased mRNA expression of *ACCN4* and *GPR65* [137]. They concluded that bone metastasis-associated mesenchymal cells have mechanisms in place to perceive the acidification of the metastasis microenvironment, leading to pain and that such findings may have implications for breast cancer palliative care [137]. The authors did not, however, establish whether ASIC4 sensed the EA, either alone or in conjunction with GPR65. In contrast to the study by Di Pompo et al. [137], Braakhuis et al. [135] noted that *ACCN4*-expression was downregulated in metastasized head-and-neck squamous-cell carcinoma compared to non-metastasized tissue.

*ACCN4*-upregulation led to improved survival outcomes in OAC in the present study. This is particularly interesting as the gene family has an established link to acid-sensing [90, 132]. Relative to other ASICs, however, literature on *ACCN4*-function is scarce. Zhou et al. [138] examined the molecular effects of *ACCN1*, *2*, *3*, and *4* and noted that only ASIC2 (encoded by *ACCN1*) promoted invasion and metastasis of colorectal cancer, under acidosis; such metastasis was achieved by the activation of the calcineurin/NFAT1 axis [8]. The subtype of the calcineurin gene protein phosphatase 3 catalytic subunit alpha isoform (*PPP3CA*) and the *NFATC1* gene was upregulated only in the OCCAMS dataset in the present study. Zhang et al. [139] showed that ASIC1 channels promote the growth of gastric cancer by upregulating autophagy. It would be interesting to expand on this study and investigate the potential effects of other ASICs on autophagy in cancer.

TRPM5 encodes a voltage-sensitive monovalent cation-selective channel, which is activated by elevated Ca^2+^ [91, 124]. EA can also influence *TRPM5*-activity [140]. Decreases in extracellular pH either quickly block *TRPM5*-induced current (IC_50_ at pH 6.2) or slowly enhance current inactivation [140]; the former is reversible while the latter is irreversible [140]. Our findings show that high *TRPM5*-expression was associated with improved survival outcomes in OAC. *TRPM5*-expression was upregulated compared to normal tissue in both the UALCAN and OCCAMS datasets. It was also upregulated in various OAC tumor grades and nodal metastatic stages in the OCCAMS dataset only. There are no studies in the literature examining the role of *TRPM5* in OAC. In an mRNA-expression analysis of bladder carcinoma patients, Ceylan et al. [141] reported significant reductions in *TRPM5*-expression in patient tissue. *TRPM5* was hypomethylated in OSCC compared to normal tissue [142]; whether such hypomethylation led to increased *TRPM5*-mRNA expression was not reported. When we examined the UALCAN portal for *TRPM5*-expression in OSCC tumor tissue, we noted no significant difference, compared to normal tissue. In a pan-cancer analysis by Qin et al. [143], no significant differences in *TRPM5* transcripts were observed between normal tissue and breast cancer, lung cancer, and colorectal cancer samples. In a hospital-based case-control study on nucleotide polymorphisms in childhood leukemia, it was observed that patients with the CG or GG genotype of the rs2301696 location in *TRPM5* had a decreased risk of developing childhood leukemia, compared to those with the CC genotype [144]. One study examined the association of *TRPM5* with patient survival in various cancers. In contrast to our findings in OAC, high *TRPM5*-mRNA expression correlated with poor overall survival in patients with melanoma and gastric cancer [50]; high *TRPM5*-expression did not, however, correlate with poor, overall survival in patients with ovarian, lung, breast, or rectal cancer [50]. In agreement with our findings from the OCCAMS dataset, *TRPM5*-expression has been implicated in metastasis [50, 51]. Sutoo et al. [51] noted that the adaptation of lung cancer cells to chronic acidic extracellular conditions (pH 6.2) elicited a sustained increase in lung cancer cell invasion and metastasis, with *TRPM5* being expressed in these cells. Similarly, in murine B16-BL6 melanoma cells, *TRPM5* mediated acidic extracellular-pH signalling, whereas *TRPM5* inhibition reduced spontaneous metastasis in these cells [50].

In the current study, *TRPM5*-upregulation was associated with improved survival outcomes. Literature citing a mechanism underlying the association of *TRPM5* with cancer is scarce. The literature which does exist focuses on *TRPM5*-related mechanisms in the tumor microenvironment (TME), or in cancer metastasis [50, 145, 146]. Mucin, secreted by goblet cells, is needed to form a physical barrier to protect epithelial cells from stress-induced damage (including acid-induced damage). Mitrovic et al. [146] concluded that, in a human colonic cancer goblet cell line, *TRPM5* mediated (via the NCX1) the entry of Na^+^ to the cell; this, in turn, resulted in the uptake of Ca^2+^ and the secretion of mucin 5AC. The upregulation of *TRPM5* in OAC tissue in the present study therefore might protect the lining of the esophagus following exposure to EA. Sakaguchi et al. [145] demonstrated a role for *TRPM5* in immune cells: they showed that *TRPM5* negatively regulated Ca^2+^-dependent inflammatory responses [production of IL-6 and chemokine C-X-C ligand 10 (CXCL10)] in B lymphocytes. Some authors have already described the role of immune cells in OAC [147–149]. Again, the upregulation of *TRPM5* observed in the present study may protect the esophagus from deleterious inflammatory responses. Maeda et al. [50] established a pathway for *TRPM5*-mediated, lung metastasis in a murine melanoma cell line: following activation by EA, *TRPM5* increased [Ca^2+^]_c_; this, in turn, activated nuclear factor kB (NF-kB) which subsequently increased the expression of matrix metalloproteinase-9. Matrix metalloproteinase-9 potentially supports the lung metastasis in this cell line, by degrading collagen in the extracellular matrix [50]. Further study in human tissue is needed to verify these findings.

*ATP2C2* encodes an ATPase pump involved in the transport of Ca^2+^ and Mn^2+^ into the Golgi [92]. It is also involved in Ca^2+^ signalling independent of its ATPase activity [92]. In particular, SPCA2 (the protein encoded by the *ATP2C2* gene) interacts with the SOCE channel, Orai1, and induces Ca^2+^ influx at the cell surface [150]. It has been shown that unbalanced, store-independent Ca^2+^ signalling can lead to enhanced cell proliferation and tumorigenesis [150]. Our findings show that *ATP2C2*-expression was upregulated in OAC in both datasets. In addition, higher *ATP2C2*-expression was associated with improved patient survival. Further analysis revealed that *ATP2C2* was significantly upregulated in various OAC tumor grades and nodal-metastatic stages in both datasets. *ORAI1* (encoding the Orai1 channel) is upregulated in OAC tissue compared to normal tissue in both the UALCAN and OCCAMS datasets, supporting this potential store-independent interaction [150]. Our *ATP2C2*-expression analysis results are consistent with those of Hyland et al. [151], who analyzed gene expression in BO. Comparison of BO-samples to same-patient, normal mucosa from squamous esophagus revealed a 2.33-fold increase in *ATP2C2* expression in BO [151]. Similar observations have been made in breast cancer analyses. In one breast cancer study, *ATP2C2* was upregulated compared to normal tissue [152]. In a separate set of breast cancer samples, SPCA2 knockdown enhanced sensitivity to DNA-damaging agents, including doxorubicin, cisplatin, and ionizing radiation [153]. Survival-related observations in the literature contradict our findings. A gene-expression study [152] noted that high *ATP2C2*-expression was associated with worsened patient survival in OSCC, breast cancer, thyroid carcinoma, head-and-neck squamous cell carcinoma, kidney, renal clear cell carcinoma, and lung squamous cell carcinoma [152]. Liu et al. [152] showed that *ATP2C2*-expression negatively correlated with patient survival in breast cancer. In a study by Makena et al. [153] on the SPCA2 protein, high abundance was associated with poor prognosis in luminal ER^+^/PR^+^ breast cancer subtypes. Similarly, Zhao et al. [154] (TCGA data) revealed a 76% decreased survival rate among thyroid cancer patients with the *ATP2C2* gene, compared to those without. Again, in contrast to our OAC findings, Zhang et al. [155] noted that the long-non-coding-RNA version of *ATP2C2* (ATP2C2-antisense 1) was associated with worsened overall survival in thyroid carcinoma. Metastasis-related studies show inconsistent results. Similar to our findings, a breast cancer analysis by Liu et al. [152] showed that *ATP2C2*-expression was correlated with advanced breast cancer stages (the “T” and “N” components). A separate, breast cancer study, however, showed that high SPCA2 levels protected against the initiation of the epithelial-mesenchymal transition [156].

*ATP2C2* upregulation was associated with improved patient survival in the present study. There is a lack of data in the literature highlighting improved patient survival with *ATP2C2*-upregulation, for any cancer. The literature on carcinogenesis points to either an Orai1-related mechanism, extracellular signal-regulated kinase 1/2 (ERK1/2)-activation, the adaptation to hypoxia, or to the influence of *ATP2C2* on the TME [92, 152, 157–159]. In OSCC, tumors displayed an increased abundance of the Orai1 protein, and this increased abundance was associated with poor overall and recurrence-free survival; furthermore, pharmacological antagonists of Orai1 reduced OSCC proliferation, invasion, and tumorigenesis [157]. Kohn et al. [158] noted that high SPCA2-abundance was correlated with epithelial genes in cancer cell lines. Similarly, Feng et al. [92] demonstrated that SPCA2-overexpression conferred increased proliferation in a nonmalignant mammary, epithelial cell line. The authors demonstrated that this increased proliferation was due to the activation of the ERK1/2 pathway [92]. Jenkins et al. [159] showed that *ATP2C2* helped colon cancer cells adapt to hypoxia, prevented cell death, increased proliferation capacity, and promoted tumor growth. Liu et al. [152] demonstrated that *ATP2C2* might be a potential indicator of TME status. Specifically, genes from the patient group with low *ATP2C2*-expression were significantly enriched in immune-related activities. Genes from the high, *ATP2C2*-expression group were mainly enriched in metabolic pathways [152]. The low, *ATP2C2*-expression group had increased numbers of pro-tumor M2 macrophages and decreased numbers of anti-tumor M1 macrophages [152]. The immune cell profile of the TME (of breast cancer in the study by Liu et al. [152]) may therefore override the tumorigenic effects inside the cell and warrants further study. Similarly, the acidic TME of OAC warrants further study and may explain improved survival in OAC patients and not in other cancers.

*GRIN2D* encodes the 2D subunit of the NMDAR. NMDARs are ligand-gated, glutamate receptors involved in Ca^2+^ signalling, primarily in neurons [160]. Our data shows that *GRIN2D*-expression was upregulated in OAC: the gene is the highest weighted-ranking gene in the two datasets examined. Zhang et al. [161] showed that *GRIN2D*-expression was upregulated in 5 OSCC samples from Chinese patients. Conversely, *GRIN2D* was among the 13 genes that were hypermethylated (downregulated) in both OAC and OSCC [162]. In meibomian cell carcinoma (cancer of the glands of the eyelids), *GRIN2D*-expression was also downregulated, compared to normal tissue [163]. In a study of various cancer cell types by Luksch et al. [164], knockdown of *GRIN2D* did not influence phenotype. There are no reported studies demonstrating a role for *GRIN2D* in patient survival. The NMDAR-2D subunit has been shown to play a role in breast-to-brain metastasis [165]. Endothelial *GRIN2D* has been shown to promote angiogenesis in colorectal cancer [166].

*TRPC4* encodes a voltage- and ligand-gated cation channel, which alters enzymatic activity and initiates endocytosis and exocytosis [167]. *TRPC4* channel activity is potentiated by decreases in pH [168]. Our data shows that TRPC4-expression was upregulated in OAC relative to normal tissue: this gene has the second-highest weighted ranking for expression (TPM) in our study. There is currently no reported evidence linking *TRPC4* with OAC. Zhang et al. [169] found in a study of 2,433 cases and 2,433 controls, that the *TRPC4* polymorphisms rs9547991 and rs978156 were candidate susceptibility markers for lung cancer in a Chinese population. Subjects carrying at least one variant allele had a 1.29-fold increased risk of developing lung cancer, compared with those carrying no variant alleles [169]. By contrast, TRPC4 has been shown to inhibit the proliferation of renal cell carcinoma cells, after the cells were exposed to englerin A (an anti-cancer substance, found in the bark of the *Phyllanthus engleri* tree) [170]. Consistent with our findings, there are no data in the literature to suggest a role for *TRPC4* in patient survival. *TRPC4*-activation by *GPR68* agonists promoted the invasion and metastasis of granule precursor-derived human medulloblastoma [171]. *TRPC4*-downregulation has been proposed as a trigger for tumor angiogenesis in renal cell carcinoma [172].

*TRPM2* encodes a Ca^2+^-permeable non-selective cation channel which is activated by adenosine diphosphate ribose (ADP-ribose), increased temperature, oxidative stress, and Ca^2+^ [173–175]. TRPM2 gating is inhibited at pH 5.5 to 6.7, indicating a role in acid-sensing [176]. Our data shows that *TRPM2* expression was upregulated in OAC: the gene has the third-highest weighted rank. There is no evidence linking a role for *TRPM2* in OAC; evidence for a role of *TRPM2* in other cancers is more extensive. A study of TRP-family-mRNA expression by Qin et al. [143], revealed that *TRPM2*-expression was upregulated in breast cancer (ductal carcinoma and invasive breast cancer), small-cell, lung carcinoma, colorectal cancer (colon and caecum adenocarcinoma), gastric cancer, and melanoma. Conversely, *TRPM2*-expression was downregulated in prostate cancer and in the brain and central nervous system cancers [143]. Sumoza-Toledo et al. [177] noted somewhat similar results in breast cancer: *TRPM2* was upregulated in invasive breast carcinoma, compared to normal tissue. *TRPM2*-expression was upregulated in oral squamous cell carcinoma (OrSCC) tissue [178]. Findings from an *in vitro* study [179] on prostate cancer contradict those from Qin et al. [143]: here, *TRPM2* played a key role in prostate cancer proliferation, as demonstrated by small interfering RNA techniques [179]. In non-small-cell, lung cancer, the long non-coding RNA [TRPM2-antisense RNA (AS)] was upregulated, and subsequent downregulation of *TRPM2* promoted apoptosis *in vitro* [180]. In OrSCC, low *TRPM2*-expression was associated with poorly- or moderately-differentiated, tumor tissue grades [181]. *TRPM2*-expression also predicted survival outcomes in breast, lung, or colorectal cancers [143]. In contrast to Qin et al. [143], a study by Sumoza-Toledo et al. [177] demonstrated that high expression was associated with improved survival outcomes in both the human epidermal growth factor receptor 2 (HER2)^+^ and ER, breast cancer subtypes. In contrast to findings reported by Chen et al. [182], Gil-Kluick et al. [183] reported that high *TRPM2* expression was associated with improved patient survival in acute myeloid leukemia (AML). Several mechanisms underlying TRPM2 function have been cited. Chen et al. [182] showed that *TRPM2* promoted AML proliferation and cellular survival through the modulation of mitochondrial function, ROS, and autophagy. In both *in vitro* and murine model studies, Almasi et al. [184] suggested that *TRPM2* promoted gastric-cancer migration, invasion, and tumor growth through the AKT pathway; the same group previously demonstrated that *TRPM2* promoted gastric-cancer cell survival via the c-Jun NH2-terminal kinase (JNK) pathway [185]. Two studies examined the role of *TRPM2* in pancreatic ductal carcinoma: the first observed that *TRPM2*-overexpression promoted cell proliferation, invasion, and migration [186]; the second implicated the protein kinase C/mitogen activated protein kinase pathway as the mechanism by which TRPM2 exerts these effects [187]. *TRPM2* was also shown to be essential for the survival and migration of OrSCC [178].

The present study has several limitations. Our analysis is based exclusively on transcriptomic data: this does not account for potential changes in protein function, resulting from altered translational or post-translational mechanisms. Evidence from an ovarian-cancer xenograft model, however, has indicated that the correlation between mRNA and protein abundance is closer for differentially expressed genes than for those whose expression is not altered [188]. A comparative analysis, using a proteomic dataset, would make the results of the present study more robust. The use of NAT, as a source of non-cancerous cells in UALCAN, is also a potential source of error: these cells might potentially be in a cancerous or pre-cancerous state [189]. Indeed, Aran et al. [189] noted that NAT may have properties distinguishing it from both tumor tissue and a more stringent classification of normal tissue; none of the 18 genes highlighted in the study were investigated in the present study. The statistical power of each dataset has an influence on the results obtained. The UALCAN portal compares data from 89 OAC-tumor samples with 11 NAT samples, while the OCCAMS dataset compares data from 213 samples with 15 normal-tissue samples. We note that none of the 21 survival-associated genes from the OCCAMS dataset were significant in UALCAN, after adjustment. We also note that a higher proportion (67.2%) of OCCAMS, heatmap-related *t*-tests proved significant after adjustment for MOT, than in UALCAN (42.9%). It is possible that the UALCAN portal did not have sufficient power to detect all statistically significant differences.

In conclusion, the present study has implicated 6 genes (*CACNA1D*, *CACNA2D4*, *JPH1*, *ACCN4*, *TRPM5*, and *ATP2C2*) as potential prognostic markers and 3 genes (*GRIN2D*, *TRPC4*, and *TRPM2*) as candidate diagnostic markers for OAC. Of these, *ACCN4*, *TRPM5*, *TRPC4*, and *TRPM2* have established roles in acid-sensing, with ACCN4 having an indirect role [140, 168, 176, 190]. With the exception of *CACNA1D* (OAC-expression data), *ATP2C2* (BO-expression data), and *GRIN2D* (OAC-methylation data), published, OAC-related data is lacking for the genes highlighted by the current study. Higher expression of all 6 prognostic genes in this study was associated with improved survival outcomes. These positive survival observations were noted in conjunction with high expression of all of these 6 genes in OAC tissue, compared to normal tissue, and sometimes (as in the case of *CACNA1D*, *JPH1*, and *ATP2C2*) in advanced metastatic stages and tumor grades. We hypothesize that these genes may be either increasing tumor cell death and/or inhibiting other cancer hallmarks, either directly or by indirect mechanisms. In the literature, the association of *CACNA1D* with improved survival outcomes in colon adenocarcinoma, and of *JPH1* with improved survival outcomes in small-cell lung carcinoma, was particularly strong [108, 127]. The associations of the other genes with survival outcomes in the literature were either inconsistent with those of our study (*CACNA2D4*, *TRPM5*, and *ATP2C2*) or unreported (*ACCN4*). With the exception of *JPH1* (targeted by miR-145 to favor tumor-suppression), *CACNA2D4* (mitigation of the adverse effects of chemotherapy in BRD9, gastric cancer), and *TRPM5* (the Ca^2+^-dependent regulation of inflammatory responses and the production of mucin), there are no clear molecular mechanisms cited to explain the improved patient survival observations in the present study. Indeed, most mechanisms cited in other cancers would intuitively lead to worsened patient survival. Because an acidic environment is what differentiates OAC from many cancers, the potential role of EA in OAC is again brought into focus. Three studies have implicated *TRPM2* in metastasis. Other studies also consistently linked high expression of *CACNA1D*, *CACNA2D4*, *JPH1*, *TRPM5*, *ATP2C2*, *GRIN2D*, and *TRPC4* with metastasis. Further research is required to discern which of these 9 genes could prove useful in OAC prognosis or diagnosis. Given the ubiquitous expression of *GRIN2D* in OAC and OSCC, *TRPC4* and *TRPM2* may be more selective, diagnostic markers for OAC. The potential interaction between the following partners in OAC may yield promising results: *CACNA1D* with Ca_v_ subunits, the RyR1 and the G protein-coupled estrogen receptor; *JPH1* and miR-145; *ACCN4* and *GPR65*; *TRPM5* and *NCXs*; *ATP2C2* and *ORAI1*; and *TRPC4* and *GPR68*. The role of the TME (particularly acidic and immune components) should also be considered in the design of such experiments. Taken together, the present study lays the groundwork for further exploration of potentially oncogenic and protective Ca^2+^-signalling pathways in OAC.

## Abbreviations

ACCN4: acid-sensing ion channel 4
ACTN: actinin α
ANOVA: analysis of variance
ASICs: acid-sensing ion channels
ATP2A: sarco-endoplasmic-reticulum Ca^2+^ ATPase
ATP2B: plasma membrane calcium ATPases
ATP2C2: secretory pathway Ca^2+^ ATPase 2
BO: Barrett’s esophagus
Ca^2+^: calcium
CACNA1D: voltage-gated Ca^2+^ channel subunit α 1D
CACNA2D4: voltage-gated Ca^2+^ channel auxiliary subunit α2 δ4
CACNB4: voltage-gated Ca^2+^ channel, L-type, β 4 subunit
CAs: carbonic anhydrases
CHRNA: nicotinic acetylcholine receptors α
EA: extracellular acid
ER: endoplasmic reticulum
FKBP: FK506-binding protein
GORD: gastroesophageal reflux disease
GPR: G-protein coupled receptors
GRIN2D: *N*-methyl-*D*-aspartate receptor 2D
GRPs: ground rubber particles
IL-1: interleukin-1
IP_3_: inositol 1,4,5-trisphophate
IP_3_Rs: inositol 1,4,5-trisphophate receptors
ITPR: inositol 1,4,5-trisphophate receptor gene
JPH1: junctophilin 1
MCOLN1: mucolipin 1
MOT: multiplicity of testing
NAT: normal-adjacent tissue
NCXs: Na^+^/Ca^2+^ exchangers
NFAT: nuclear factor of activated T cells
NMDARs: *N*-methyl-*D*-aspartate receptors
OAC: oesophageal adenocarcinoma
OCCAMS: Oesophageal Cancer Clinical and Molecular Stratification
OrSCC: oral squamous cell carcinoma
OSCC: oesophageal squamous cell carcinoma
PKD: polycystin
PM: plasma membrane
ROS: reactive oxygen species
RyRs: ryanodine receptors
SERCAs: sarcoplasmic reticulum/endoplasmic reticulum Ca^2+^-ATPases
SLC24A: sodium-potassium-Ca^2+^ exchanger
SLC26: solute-carrier family 26 member
SLC4A: solute-carrier family 4 member A
SLC8A: sodium-Ca^2+^ exchanger
SLC9A: Solute Carrier 9A
SOCE: store-operated Ca^2+^-entry
SPCA: secretory pathway calcium ATPase
STIM: stromal interaction molecule
STOML: stomatin-like
TCGA: The Cancer Genome Atlas
TGF-β: transforming growth factor-beta
TME: tumor microenvironment
TPM: transcripts per million
TRP: transient receptor potential
TRPC4: transient receptor potential ion channel classical or canonical 4
TRPM2: transient receptor potential ion channel subfamily M member 2
TRPV6: transient receptor potential vanilloid 6
VGCCs: voltage-gated Ca^2+^ channels

## Supplementary materials

The supplementary material for this article is available at: https://www.explorationpub.com/uploads/Article/file/100263_sup_1.pdf.

## Declarations

### Availability of data and materials

All data used in this manuscript were obtained from either The Cancer Genome Atlas (https://www.cancer.gov/about-nci/organization/ccg/research/structural-genomics/tcga), accessed via the UALCAN portal (http://ualcan.path.uab.edu/cgi-bin/ualcan-res.pl/), or the Oesophageal Cancer Clinical and Molecular Stratification (OCCAMS) dataset (https://www.mrc-cu.cam.ac.uk/research/rebecca-fitzgerald/clinical-studies/occams).

### Copyright

© The Author(s) 2021.
