# Supplementary material for: Alterations in the Ca2+ toolkit in oesophageal adenocarcinoma
Source: Explor Target Antitumor Ther. 2021 Dec 31;2(6):543–75. doi: 10.37349/etat.2021.00063 (PMC9400700; doi:10.37349/etat.2021.00063)
Supplement: Supplementary file 1 [file etat-02-100263-s001.pdf]

SUPPLEMENTARY MATERIAL:

**Supplemental Table S1. List of 275 Ca^{2+}-Toolkit Genes Analysed in both the UALCAN and OCCAMS Datasets**	
**Gene:**	**Protein Name:**
**ACCN1**	Acid-sensing Ion Channel 2
**ACCN2**	Acid-sensing Ion Channel 1
**ACCN3**	Acid-sensing Ion Channel 3
**ACCN4**	Acid-sensing Ion Channel 4
**ACTN1**	Actinin α 1
**ACTN2**	Actinin α 2
**ACTN3**	Actinin α 3
**ACTN4**	Actinin α 4
**AHCYL1**	Adenosylhomocysteinase-Like 1
**AKAP5**	A-Kinase Anchoring Protein 5
**AKAP6**	A-Kinase Anchoring Protein 6
**AKAP9**	A-Kinase Anchoring Protein 9
**ANXA1**	Annexin A1
**ANXA10**	Annexin A10
**ANXA11**	Annexin A11
**ANXA13**	Annexin A13
**ANXA2**	Annexin A2
**ANXA3**	Annexin A3
**ANXA4**	Annexin A4
**ANXA5**	Annexin A5
**ANXA6**	Annexin A6

**ANXA7**	Annexin A7
**ANXA8**	Annexin A8
**ANXA9**	Annexin A9
**ASPH**	Aspartate β-Hydroxylase
**ATF4**	Activating Transcription Factor 4
**ATP2A1**	Sarco-endoplasmic-reticulum Ca^{2+} ATPase 1
**ATP2A2**	Sarco-endoplasmic-reticulum Ca^{2+} ATPase 2
**ATP2A3**	Sarco-endoplasmic-reticulum Ca^{2+} ATPase 3
**ATP2B1**	Plasma Membrane Ca^{2+} ATPase 1
**ATP2B4**	Plasma Membrane Ca^{2+} ATPase 4
**ATP2C1**	Secretory Pathway Ca^{2+} ATPase 1
**ATP2C2**	Secretory Pathway Ca^{2+} ATPase 2
**ATP6V0A1**	ATPase H^+ Transporting V0 Subunit A1
**ATP6V0A2**	ATPase H^+ Transporting V0 Subunit A2
**ATP6V0A4**	ATPase H^+ Transporting V0 Subunit A4
**ATP6V0B**	ATPase H^+ Transporting V0 Subunit B
**ATP6V0C**	ATPase H^+ Transporting V0 Subunit C
**C22ORF32**	Essential MCU Regulator
**CA1**	Carbonic Anhydrase 1
**CA10**	Carbonic Anhydrase 10
**CA11**	Carbonic Anhydrase 11
**CA12**	Carbonic Anhydrase 12
**CA13**	Carbonic Anhydrase 13
**CA14**	Carbonic Anhydrase 14
**CA2**	Carbonic Anhydrase 2

**CA3**	Carbonic Anhydrase 3
**CA4**	Carbonic Anhydrase 4
**CA6**	Carbonic Anhydrase 6
**CA7**	Carbonic Anhydrase 7
**CA8**	Carbonic Anhydrase 8
**CA9**	Carbonic Anhydrase 9
**CACNA1A**	Voltage-Gated Ca^{2+} Channel, P/Q Type, A 1A Subunit.
**CACNA1B**	Voltage-Gated Ca^{2+} Channel, N-Type, A 1B Subunit.
**CACNA1C**	Voltage-Gated Ca^{2+} Channel, L-Type, A 1C Subunit.
**CACNA1D**	Voltage-Gated Ca^{2+} Channel, L-Type, A 1D Subunit.
**CACNA1H**	Voltage-Gated Ca^{2+} Channel, T-Type, A 1H Subunit.
**CACNA2D1**	Voltage-Gated Ca^{2+} Channel Auxiliary Subunit A 2 Δ 1
**CACNA2D2**	Voltage-Gated Ca^{2+} Channel Auxiliary Subunit A 2 Δ 2
**CACNA2D3**	Voltage-Gated Ca^{2+} Channel Auxiliary Subunit A 2 Δ 3
**CACNA2D4**	Voltage-Gated Ca^{2+} Channel Auxiliary Subunit A 2 Δ 4
**CACNB1**	Voltage-Gated Ca^{2+} Channel, L-Type, β 1 Subunit
**CACNB2**	Voltage-Gated Ca^{2+} Channel, L-Type, β 2 Subunit
**CACNB3**	Voltage-Gated Ca^{2+} Channel, L-Type, β 3 Subunit
**CACNB4**	Voltage-Gated Ca^{2+} Channel, L-Type, β 4 Subunit
**CACNG4**	Voltage-Gated Ca^{2+} Channel Auxiliary Subunit Gamma 4
**CALB1**	Calbindin 1
**CALB2**	Calbindin 2
**CALM1**	Calmodulin 1
**CALM2**	Calmodulin 2
**CALM3**	Calmodulin 3

**CALR**	Calreticulin
**CAMK1**	Calmodulin-dependent Protein Kinase 1
**CAMK1D**	Calmodulin-dependent Protein Kinase 1D
**CAMK2A**	Calmodulin-dependent Protein Kinase 2A
**CAMK2B**	Calmodulin-dependent Protein Kinase 2B
**CAMK2D**	Calmodulin-dependent Protein Kinase 2D
**CAMK4**	Calmodulin-dependent Protein Kinase 4
**CANX**	Calnexin
**CASQ2**	Calsequestrin 1
**CBARA1**	Mitochondrial Ca^{2+} Uptake 1
**CCDC109A**	Mitochondrial Ca^{2+} Uniporter
**CCDC90A**	Mitochondrial Ca^{2+} Uniporter Regulator 1
**CD38**	Cluster of Differentiation 38
**CHRNA1**	Nicotinic Cholinergic Receptor A1
**CHRNA10**	Nicotinic Cholinergic Receptor A10
**CHRNA3**	Nicotinic Cholinergic Receptor A3
**CHRNA5**	Nicotinic Cholinergic Receptor A5
**CHRNA7**	Nicotinic Cholinergic Receptor A7
**CHRNA9**	Nicotinic Cholinergic Receptor A9
**CNGA1**	Cyclic Nucleotide Gated Channel Subunit α 1
**CNGB1**	Cyclic Nucleotide Gated Channel Subunit β 1
**CREB1**	CAMP Responsive Element Binding Protein 1
**CREB3**	CAMP Responsive Element Binding Protein 3
**CREB3L1**	CAMP Responsive Element Binding Protein 3-Like 1
**CREB3L2**	CAMP Responsive Element Binding Protein 3-Like 2

**CREB3L3**	CAMP Responsive Element Binding Protein 3-Like 3
**CREB3L4**	CAMP Responsive Element Binding Protein 3-Like 4
**CREB5**	CAMP Responsive Element Binding Protein 5
**EFHA1**	Mitochondrial Ca^{2+} Uptake 2
**EFHA2**	Mitochondrial Ca^{2+} Uptake Family Member 3
**FKBP1A**	12 kDa FK506-Binding Protein
**FKBP1B**	12.6 kDa FK506-Binding Protein
**GNA11**	G Protein Subunit α 11
**GNA14**	G Protein Subunit α 14
**GNAQ**	G Protein Subunit α Q
**GNB1**	G Protein Subunit β 1
**GNB2**	G Protein Subunit β 2
**GNB3**	G Protein Subunit β 3
**GNB4**	G Protein Subunit β 4
**GNB5**	G Protein Subunit β 5
**GNG10**	G Protein Subunit Gamma 10
**GNG11**	G Protein Subunit γ 11
**GNG12**	G Protein Subunit γ 12
**GNG2**	G Protein Subunit γ 2
**GNG3**	G Protein Subunit γ 3
**GNG4**	G Protein Subunit γ 4
**GNG5**	G Protein Subunit γ 5
**GNG7**	G Protein Subunit γ 7
**GOPC**	Golgi-Associated PDZ and Coiled-coil Motif Containing
**GPR132**	G Protein-Coupled Receptor 132

**GPR4**	G Protein-Coupled Receptor 4
**GPR65**	G Protein-Coupled Receptor 65
**GPR68**	G Protein-Coupled Receptor 68
**GRIN1**	N-Methyl-D-Aspartate Receptor 1
**GRIN2A**	N-Methyl-D-Aspartate Receptor Subunit 2A
**GRIN2D**	N-Methyl-D-Aspartate Receptor Subunit 2D
**HOMER1**	Homer Scaffold Protein 1
**HOMER2**	Homer Scaffold Protein 2
**HOMER3**	Homer Scaffold Protein 3
**HSP90AA1**	Heat Shock 90kDa Protein 1, α
**HSP90AB1**	Heat Shock 90kDa Protein 1, β
**HSPA13**	Heat Shock 70kDa Protein A13
**HSPA14**	Heat Shock 70kDa Protein A14
**HSPA1A**	Heat Shock 70kDa Protein 1A
**HSPA1B**	Heat Shock 70kDa Protein 1B
**HSPA1L**	Heat Shock 70kDa Protein 1L
**HSPA2**	Heat Shock 70kDa Protein A2
**HSPA4**	Heat Shock 70kDa Protein A4
**HSPA5**	Heat Shock 70kDa Protein A5
**HSPA6**	Heat Shock 70kDa Protein A6
**HSPA7**	Heat Shock 70kDa Protein A7
**HSPA8**	Heat Shock 70kDa Protein A8
**HSPA9**	Heat Shock 70kDa Protein A9
**HVCN1**	Hydrogen Voltage Gated Channel 1
**ITPR1**	Inositol 1,4,5-Trisphosphate Receptor Type 1

**ITPR2**	Inositol 1,4,5-Trisphosphate Receptor Type 2
**ITPR3**	Inositol 1,4,5-Trisphosphate Receptor Type 3
**JPH1**	Junctophilin 1
**JPH2**	Junctophilin 2
**JPH3**	Junctophilin 3
**JPH4**	Junctophilin 4
**KCNIP2**	Potassium Voltage-Gated Channel Interacting Protein 2
**KCNIP3**	Potassium Voltage-Gated Channel Interacting Protein 3
**KCNIP4**	Potassium Voltage-Gated Channel Interacting Protein 4
**LETM1**	Mitochondrial Proton/Ca^{2+} Exchanger Protein
**MCOLN1**	Mucolipin 1
**MCOLN2**	Mucolipin 2
**MRVI1**	Murine Retrovirus Integration Site 1 Homolog
**NCS1**	Neuronal Ca^{2+} Sensor 1
**NFATC1**	Nuclear Factor of Activated T Cells 1
**NFATC2**	Nuclear Factor of Activated T Cells 2
**NFATC3**	Nuclear Factor of Activated T Cells 3
**NFATC4**	Nuclear Factor of Activated T Cells 4
**ORAI1**	Ca^{2+}-Release Activated Ca^{2+} Modulator 1
**ORAI2**	Ca^{2+}-Release Activated Ca^{2+} Modulator 2
**ORAI3**	Ca^{2+}-Release Activated Ca^{2+} Modulator 3
**P2RX7**	Purinergic Receptor P2X, Ligand Gated Ion Channel, 7
**PICK1**	Protein Interacting with C Kinase 1
**PKD1**	Polycystin 1
**PKD2**	Polycystin 2

**PLCB1**	Phospholipase C β 1
**PLCB2**	Phospholipase C β 2
**PLCB3**	Phospholipase C β 3
**PLCD1**	Phospholipase C δ 1
**PLCD3**	Phospholipase C δ 3
**PLCD4**	Phospholipase C δ 4
**PLCE1**	Phospholipase C ε 1
**PLCG1**	Phospholipase C γ 1
**PLN**	Phospholamban
**PPP1CA**	Protein Phosphatase 1 Catalytic Subunit α
**PPP2CA**	Protein Phosphatase 2 Catalytic Subunit α
**PPP3CA**	Protein Phosphatase 3 Catalytic Subunit α
**PPP3CB**	Protein Phosphatase 3 Catalytic Subunit β
**PPP3CC**	Protein Phosphatase 3 Catalytic Subunit γ
**PRKCG**	Protein Kinase C γ
**PRKCI**	Protein Kinase C ι
**PRKCZ**	Protein Kinase C ζ
**PVALB**	Parvalbumin
**RGS1**	Regulator of G Protein Signalling 1
**RGS16**	Regulator of G Protein Signalling 16
**RGS2**	Regulator of G Protein Signalling 2
**RGS4**	Regulator of G Protein Signalling 4
**RYR1**	Ryanodine Receptor 1
**RYR2**	Ryanodine Receptor 2
**RYR3**	Ryanodine Receptor 3

**S100A1**	S100 Ca^{2+} Binding Protein A1
**S100A10**	S100 Ca^{2+} Binding Protein A10
**S100A11**	S100 Ca^{2+} Binding Protein A11
**S100A12**	S100 Ca^{2+} Binding Protein A12
**S100A13**	S100 Ca^{2+} Binding Protein A13
**S100A14**	S100 Ca^{2+} Binding Protein A14
**S100A16**	S100 Ca^{2+} Binding Protein A16
**S100A2**	S100 Ca^{2+} Binding Protein A2
**S100A3**	S100 Ca^{2+} Binding Protein A3
**S100A4**	S100 Ca^{2+} Binding Protein A4
**S100A5**	S100 Ca^{2+} Binding Protein A5
**S100A6**	S100 Ca^{2+} Binding Protein A6
**S100A7**	S100 Ca^{2+} Binding Protein A7
**S100A8**	S100 Ca^{2+} Binding Protein A8
**S100A9**	S100 Ca^{2+} Binding Protein A9
**S100B**	S100 Ca^{2+} Binding Protein B
**S100P**	S100 Ca^{2+} Binding Protein P
**SEPN1**	Selenoprotein N1
**SLC24A1**	Sodium-Potassium-Ca^{2+} Exchanger 1
**SLC24A3**	Sodium-Potassium-Ca^{2+} Exchanger 3
**SLC24A5**	Sodium-Potassium-Ca^{2+} Exchanger 5
**SLC24A6**	Na(+)/K(+)/Ca(2+)-Exchange Protein 6
**SLC25A4**	ADP/ATP translocase 1
**SLC25A5**	ADP/ATP translocase 2
**SLC26A1**	Solute Carrier Family 26 Member 1

**SLC26A10**	Solute Carrier Family 26 Member 10
**SLC26A2**	Solute Carrier Family 26 Member 2
**SLC26A3**	Solute Carrier Family 26 Member 3
**SLC26A4**	Solute Carrier Family 26 Member 4
**SLC26A5**	Solute Carrier Family 26 Member 5
**SLC26A6**	Solute Carrier Family 26 Member 6
**SLC26A7**	Solute Carrier Family 26 Member 7
**SLC26A8**	Solute Carrier Family 26 Member 8
**SLC26A9**	Solute Carrier Family 26 Member 9
**SLC4A1**	Solute Carrier Family 4 Member 1 (Diego Blood Group)
**SLC4A10**	Solute Carrier Family 4 Member 10
**SLC4A11**	Solute Carrier Family 4 Member 11
**SLC4A2**	Solute Carrier Family 4 Member 2
**SLC4A3**	Solute Carrier Family 4 Member 3
**SLC4A4**	Solute Carrier Family 4 Member 4
**SLC4A5**	Solute Carrier Family 4 Member 5
**SLC4A7**	Solute Carrier Family 4 Member 7
**SLC4A8**	Solute Carrier Family 4 Member 8
**SLC4A9**	Solute Carrier Family 4 Member 9
**SLC8A1**	Sodium-Ca^{2+} Exchanger 1
**SLC8A2**	Sodium-Ca^{2+} Exchanger 2
**SLC9A1**	Sodium/Hydrogen Exchanger 1
**SLC9A2**	Sodium/Hydrogen Exchanger 2
**SLC9A3**	Sodium/Hydrogen Exchanger 3
**SLC9A4**	Sodium/Hydrogen Exchanger 4

**SLC9A5**	Sodium/Hydrogen Exchanger 5
**SLC9A6**	Sodium/Hydrogen Exchanger 6
**SLC9A7**	Sodium/Hydrogen Exchanger 7
**SLC9A8**	Sodium/Hydrogen Exchanger 8
**SLC9A9**	Sodium/Hydrogen Exchanger 9
**SRI**	Sorcin
**STIM1**	Stromal Interaction Molecule 1
**STIM2**	Stromal Interaction Molecule 2
**STOML1**	Stomatin-Like 1
**STOML2**	Stomatin-Like 2
**TPCN1**	Two Pore Segment Channel 1
**TPCN2**	Two Pore Segment Channel 2
**TRPA1**	Transient Receptor Potential Cation Channel Subfamily A Member 1
**TRPC1**	Transient Receptor Potential Ion Channel Subfamily C Member 1
**TRPC4**	Transient Receptor Potential Ion Channel Subfamily C Member 4
**TRPC6**	Transient Receptor Potential Ion Channel Subfamily C Member 6
**TRPM2**	Transient Receptor Potential Ion Channel Subfamily M Member 2
**TRPM4**	Transient Receptor Potential Ion Channel Subfamily M Member 4
**TRPM5**	Transient Receptor Potential Cation Channel Subfamily M Member 5
**TRPM6**	Transient Receptor Potential Ion Channel Subfamily M Member 6
**TRPM7**	Transient Receptor Potential Ion Channel Subfamily M Member 7
**TRPV1**	Transient Receptor Potential Ion Channel Subfamily V Member 1
**TRPV2**	Transient Receptor Potential Ion Channel Subfamily V Member 2
**TRPV3**	Transient Receptor Potential Ion Channel Subfamily V Member 3
**TRPV4**	Transient Receptor Potential Cation Channel Subfamily V Member 4

**TRPV5**	Transient Receptor Potential Cation Channel Subfamily V Member 5
**TRPV6**	Transient Receptor Potential Ion Channel Subfamily V Member 6
**VDAC1**	Voltage Dependent Anion Channel 1
**VDAC2**	Voltage Dependent Anion Channel 2
**VDAC3**	Voltage Dependent Anion Channel 3

A)

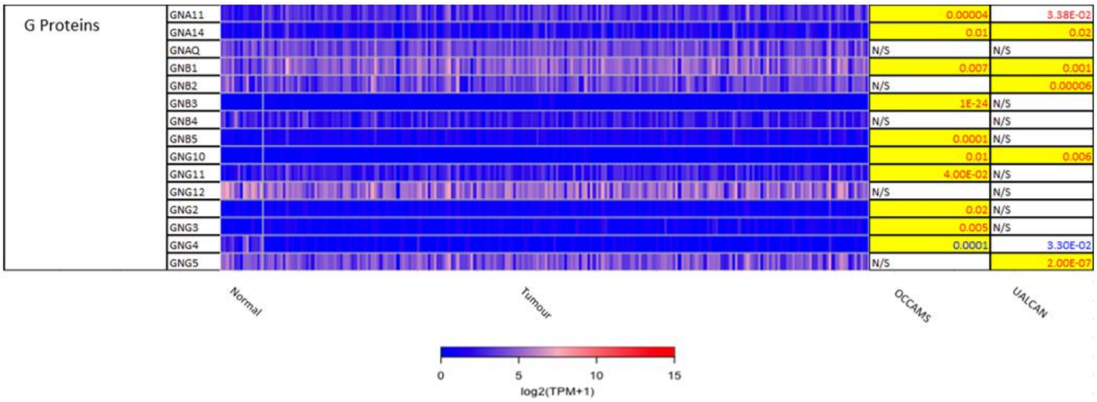

B)

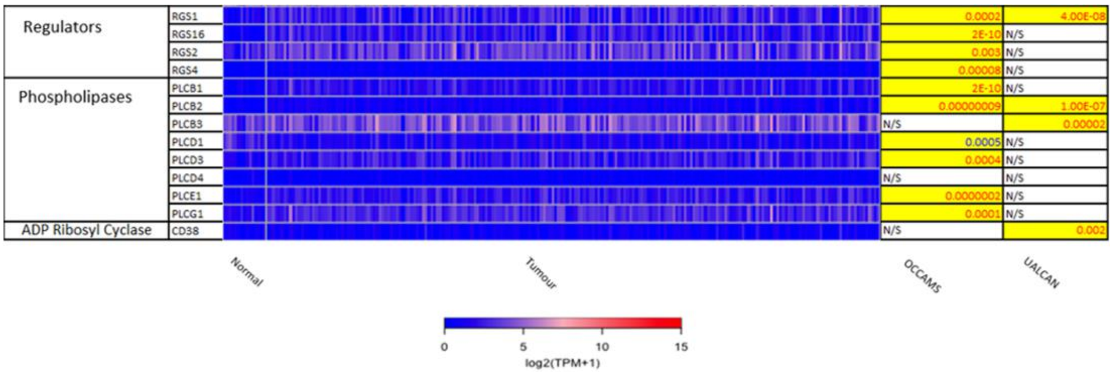

Supplemental Figure S1. Expression of Ca^{2+}-Signal Transducers in OAC. Heatmap depicting the expression-levels of genes encoding proteins involved in Ca^{2+} signal transduction: G Proteins (*Panel A*) and Regulators, Phospholipases and ADP Ribosyl Cyclase (*Panel B*). *Please see Figure 2 for details.* Expression data on GNG7 was not available from the OCCAMS dataset and was downregulated in the UALCAN data (P = 3.42e-2; not significant after adjustment for MOT).

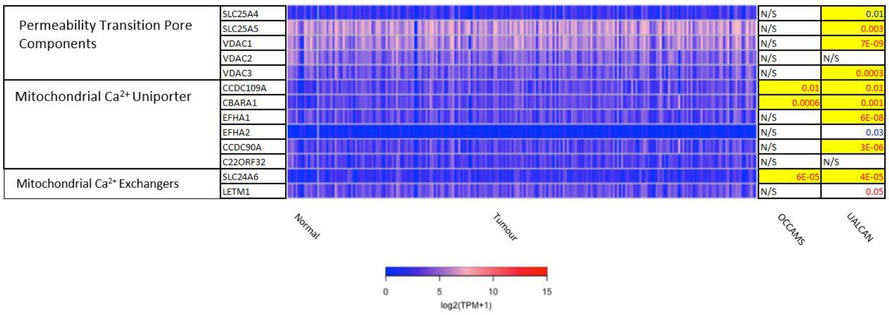

Supplemental Figure S2. Expression of Mitochondria-Associated, Ca^{2+}-Toolkit Genes in OAC. *Please see Figure 2 for details.*

A)

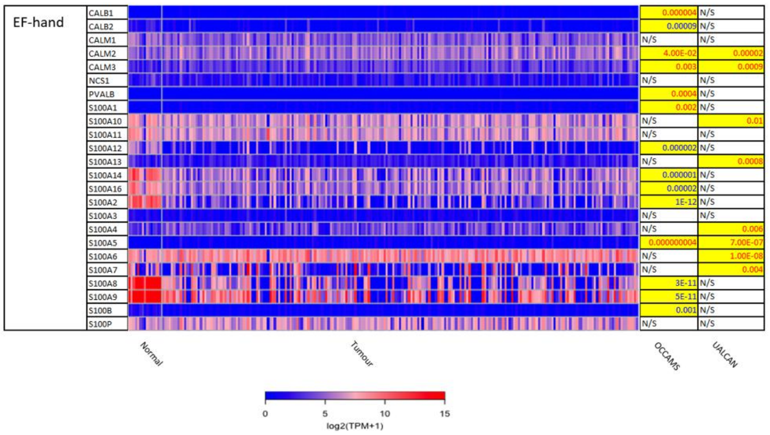

B)

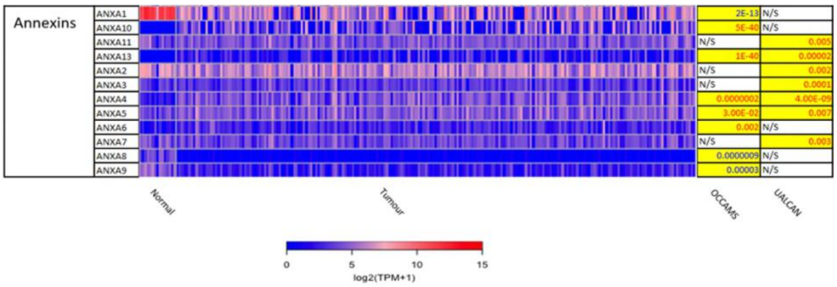

Supplemental Figure S3. Expression of Cytosolic Ca^{2+}-sensors and -buffers in OAC. *Please see Figure 2 for details.* SRI was not available in the OCCAMS data but was upregulated in the UALCAN data (P = 1e-8).

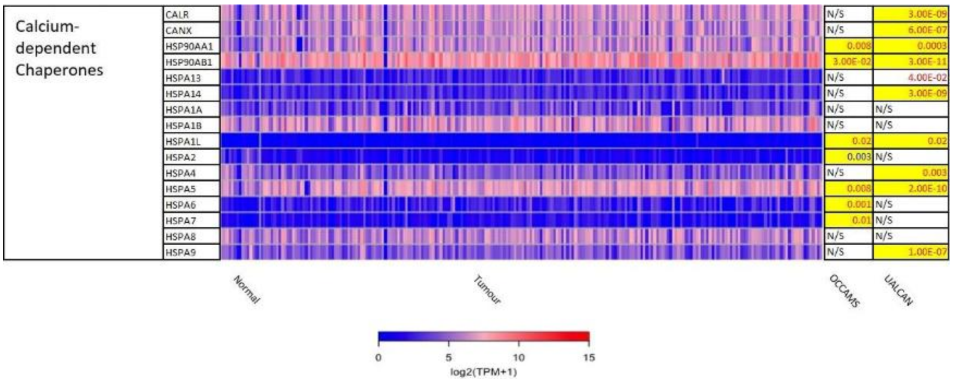

Supplemental Figure S4. Expression of Ca^{2+}-dependent Chaperones in OAC. *For details, please see Figure 2.*

A)

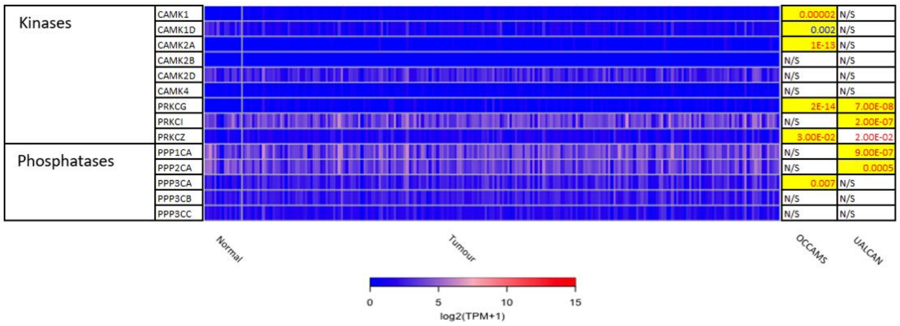

B)

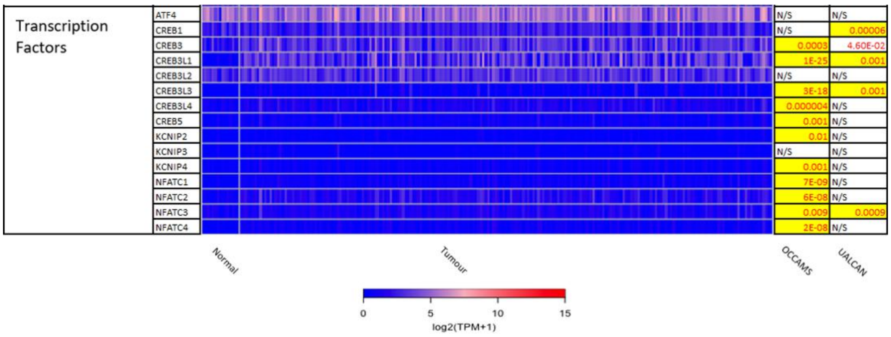

Supplemental Figure S5. Expression of Ca^{2+}-dependent effectors in OAC. *See Figure 2 for details.*

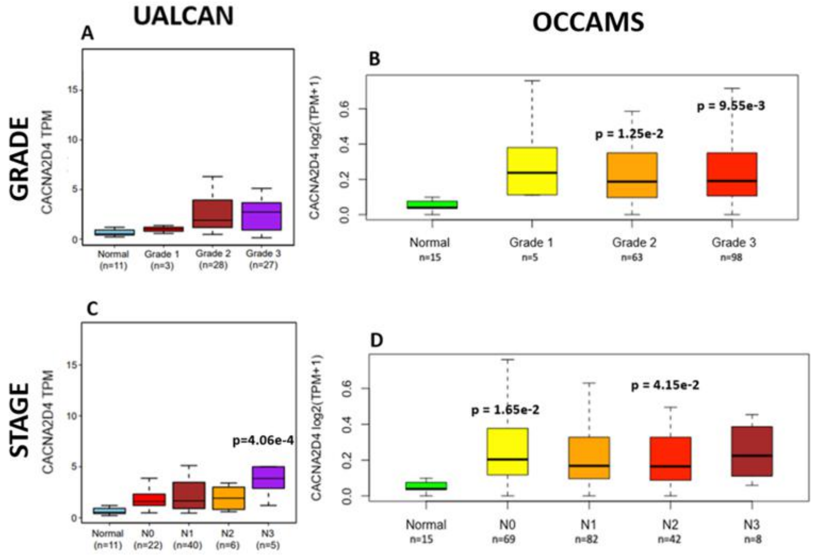

Supplemental Figure S6. OAC-Tumour, Grade and Nodal-Metastatic-Stage Boxplots for CACNA2D4. Details as described in *Figure 7.*

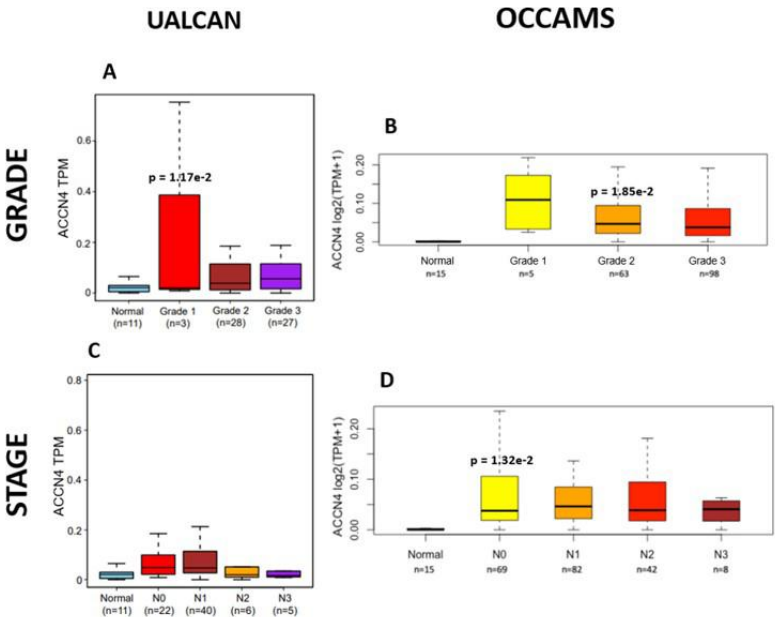

Supplemental Figure S7. OAC-Tumour, Grade and Nodal-Metastatic-Stage Boxplots for ACCN4. Details as described in *Figure 7.*

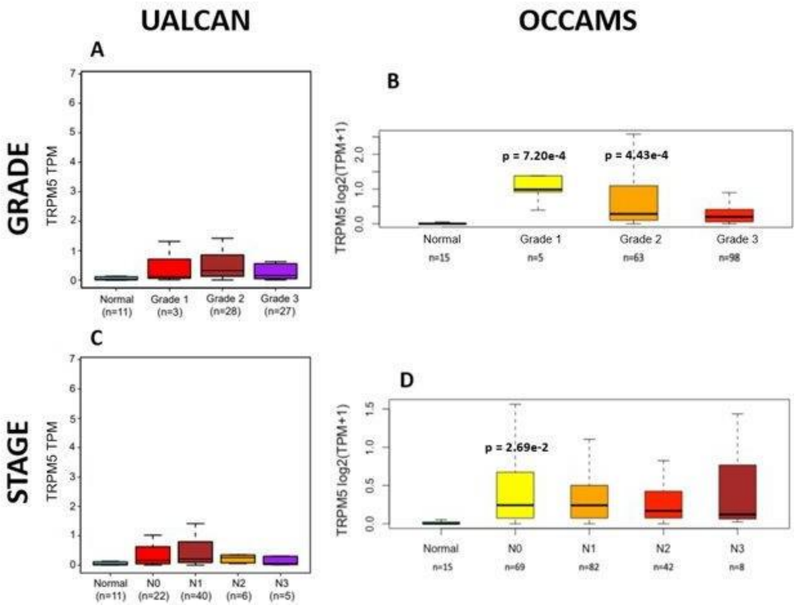

Supplemental Figure S8. OAC-Tumour, Grade and Nodal-Metastatic-Stage Boxplots for TRPM5. Details as described in *Figure 7.*
